# Pupylation‐Based Proximity Labeling Unravels a Comprehensive Protein and Phosphoprotein Interactome of the Arabidopsis TOR Complex

**DOI:** 10.1002/advs.202414496

**Published:** 2025-03-24

**Authors:** Shuai Zheng, Leonard Blaschek, Delphine Pottier, Luuk Robin Hoegen Dijkhof, Beyza Özmen, Peng Ken Lim, Qiao Wen Tan, Marek Mutwil, Alexander Sebastian Hauser, Staffan Persson

**Affiliations:** ^1^ Copenhagen Plant Science Center (CPSC) Department of Plant & Environmental Sciences University of Copenhagen Frederiksberg C 1871 Denmark; ^2^ Department of Drug Design and Pharmacology Faculty of Health and Medical Sciences University of Copenhagen Copenhagen 2100 Denmark; ^3^ School of Biological Sciences Nanyang Technological University Singapore 637551 Singapore; ^4^ Joint International Research Laboratory of Metabolic & Developmental Sciences State Key Laboratory of Hybrid Rice SJTU‐University of Adelaide Joint Centre for Agriculture and Health School of Life Sciences and Biotechnology Shanghai Jiao Tong University Shanghai 200240 China

**Keywords:** AlphaFold, proximity labeling, PUP‐IT, sugar signaling, target of rapamycin

## Abstract

Target of rapamycin (TOR) is a signaling hub that integrates developmental, hormonal, and environmental signals to optimize carbon allocation and plant growth. In plant cells, TOR acts together with the proteins LST8‐1 and RAPTOR1 to form a core TOR complex (TORC). While these proteins comprise a functional TORC, they engage with many other proteins to ensure precise signal outputs. Although TORC interactions have attracted significant attention in the recent past, large parts of the interactome are still unknown. In this resource study, PUP‐IT is adapted, a fully endogenously expressed protein proximity labeling toolbox, to map TORC protein–protein interactions using the core set of TORC as baits. It is outlined how this interactome is differentially phosphorylated during changes in carbon availability, uncovering putative direct TOR kinase targets. An AlphaFold‐Multimer approach is further used to validate many interactors, thus outlining a comprehensive TORC interactome that includes over a hundred new candidate interactors and provides an invaluable resource to the plant cell signaling community.

## Graphical abstract

The target of rapamycin (TOR) complex is a central plant growth regulator. Here, the protein–protein interactions underlying its various functions are mapped by pupylation‐based proximity‐labeling in Arabidopsis, using TOR complex subunits LST8‐1 and RAPTOR1, alongside TOR inhibitor FK506‐binding protein, as baits. The resulting interactomes from seedlings with varying sugar status will accelerate research in growth‐defense trade‐offs and sugar signaling.

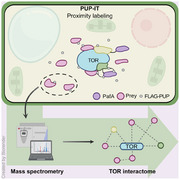

## 1 Introduction

The survival of organisms relies on their ability to sense nutrients and environmental cues to coordinate growth and development. In plants and other eukaryotes, target of rapamycin (TOR), an evolutionary conserved Ser/Thr kinase, critically regulates growth by integrating metabolic and environmental signals. TOR orchestrates its functions via a set of core interactors forming two distinct complexes in yeast and animal cells: mTORC1 and mTORC2.^[^ ^1^ ^]^ By contrast, in plants only TORC1 (hereafter TORC) has been identified, which is composed of TOR, regulatory‐associated protein of tor (RAPTOR) and lethal with sec thirteen 8 (LST8).^[^ ^2^ ^]^ The role of mTORC2 in plants might either be filled by another protein complex or by specific protein–protein interactions (PPIs) of TORC1.

In plants, TORC responds to a broad range of upstream signals, including nutrients, energy status, phytohormones, light, and stress, prompting alterations in downstream pathways, which in turn regulate cell growth and proliferation.^[^ ^2^, ^3^, ^4^ ^]^ For instance, TORC is activated by glucose, which promotes anabolic processes, such as biosynthesis of nucleotides, lipids and amino acids, photosynthesis, protein translation, and cell proliferation, while repressing catabolic processes, such as autophagy.^[^ ^2^, ^5^ ^]^ As a growth‐regulating hub, TORC signaling is also tightly connected to virtually all phytohormones including the growth‐promoting hormones auxin and brassinosteroids, as well as the growth‐inhibiting abscisic acid, jasmonic acid, and ethylene.^[^ ^2^, ^6^, ^7^ ^]^ TORC also prominently regulates protein biosynthesis. Here, TORC interacts with various ribosomal proteins along with RIBOSOMAL PROTEIN S6 KINASE 1 (S6K1), a regulator of ribosomal protein translation.^[^ ^4^, ^8^, ^9^, ^10^, ^11^ ^]^

Apart from the core TORC, numerous accessory proteins modulate TOR activity through PPIs in plants. Rho‐like small GTPase 2 (ROP2) binds to and activates TOR in response to light, auxin and nitrogen‐related nutrients, thereby regulating plant growth.^[^ ^12^, ^13^, ^14^ ^]^ The energy sensor SnRK1 (SNF1‐RELATED KINASE 1), an ortholog of mammalian AMPK α‐subunit, modulates TOR activity in response to changing energy availability. Under unfavorable conditions, such as energy deprivation or drought stress, KIN10, one of the catalytic subunits of SnRK1, directly interacts with and phosphorylates RAPTOR, resulting in TOR suppression.^[^ ^15^, ^16^ ^]^ Finally, water stress‐triggered ABA signaling causes SnRK2s to phosphorylate RAPTOR, causing the dissociation of the TORC, inhibition of TOR activity, and subsequent growth suppression.^[^ ^17^ ^]^

Overall, TORC has 66 interactors supported by different degrees of experimental evidence in Arabidopsis (*A. thaliana*), or closely related homologs in other species, as listed in StringDB. Additionally, Van Leene et al.^[^ ^11^ ^]^ identified 137 proteins (partly overlapping with the aforementioned) that interacted with TORC using pull‐down and tandem affinity purification (TAP) experiments. Recently, Persyn et al.^[^ ^18^ ^]^ mapped the interaction network downstream of TOR, using both pull‐down approaches and TurboID proximity labeling in cell cultures. While these works have unquestionably pushed the field forward, many TOR interactions likely remain unknown. Identifying these interactions, including transient interactions and those exclusive to specific plant tissues, will be crucial for our understanding of TOR signaling and regulation.

Proximity labeling based PPI approaches may identify both stable and transieXVnt PPIs. Among these, pupylation‐based proximity labeling (PUP‐IT) has emerged as a promising technique, exclusively leveraging genetically encoded components. Originally developed in mammalian cells, PUP‐IT has recently been adapted for use in plant systems.^[^ ^19^, ^20^ ^]^ PUP‐IT functions via the combined expression of a PUP(E) peptide and a protein fusion of peptide ligase PafA to the protein of interest. PPI‐mediated proximity to PafA then triggers PUP(E)‐labeling of interactors within roughly 10 nm and in the same subcellular compartment as the bait. Labeled interactors can then be identified using mass spectrometry. Here, we employed PUP‐IT to identify TORC interactors, providing an important resource to the TORC research community. Through a range of complementary constructs and treatments, we identify known but also many new TORC interactors that depend on the sugar status of the plant, and outline putative direct phospho‐targets of TORC. Our findings provide new insights into the TOR signaling pathway, which can be utilized to study carbon allocation and biomass accumulation in plants.

## 2 Results

### 2.1 Pupylation‐Based Proximity Labeling Successfully Targets TOR Complex Components

**Figure 1:**
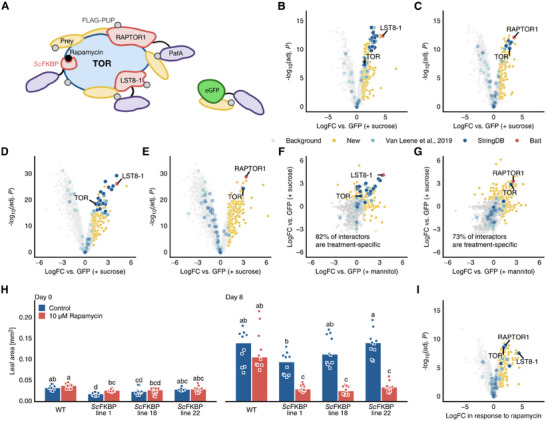
Combining different treatments and baits determines different components of the TORC interactome. A) Scheme illustrating PUP‐IT in the context of TOR signaling. PafA fused to the baits LST8‐1, RAPTOR1, and ScFKBP labels individual and TOR complex associated protein interactors, while a GFP fusion acts as a control for unspecific interactions. B–E) Volcano plots showing proteins significantly enriched in LST8‐1 B,D) and RAPTOR1 C,E) baits using constitutive B,C) or inducible D,E) FLAG::PUP(E) expression. F–G) Treatment‐dependency of identified interactors using LST8‐1 G) or RAPTOR1 H) baits and inducible FLAG::PUP(E) expression. Treatment‐specific interactors are those only identified in one of the two treatments. H) Rapamycin response of WT and *Sc*FKBP‐producing seedlings (*n* = 10 seedlings). Bar height indicates group median. I) Volcano plot showing proteins significantly enriched in the *Sc*FKBP bait using constitutive FLAG::PUP(E) expression. In all volcano plots, fold changes are calculated from *n* = 3 replicates using MsqRob2, *p*‐values are corrected for multiple comparisons using the Benjamini–Hochberg FDR method. Different letters in panel (H) indicate statistically significant differences between groups based on a one‐way ANOVA followed by a Tukey‐HSD test (𝛼 = 0.05).

To identify both constitutive and transient TORC interactors, we fused the PUP(E) ligase PafA to TORC subunits LST8‐1 and RAPTOR1, generating LST8‐1::PafA and RAPTOR1::PafA, driven by a ubiquitin10 promoter.^[^ ^8^, ^21^ ^]^ An eGFP fusion was used as a general cytosolic control (**Figure** **1**A). On the same vector backbone, we also included constitutively expressed FLAG::PUP(E) fusions driven by a 35S promoter or a 𝛽‐estradiol inducible Locus for X‐ray sensitivity A–Virion Protein 16–Estrogen Receptor (XVE) cassette. We first confirmed efficient PUP‐labeling via transient expression in *N. benthamiana* leaves, visualizing FLAG::PUP(E)‐tagged proteins by anti‐FLAG immunoblotting (Figure S1A,B, Supporting Information). After enrichment from samples with 35S driven FLAG::PUP(E), FLAG‐tagged protein was quantified by label‐free mass spectrometry (Figure S1C, Supporting Information). The results of a differential enrichment analysis revealed strong labeling of the baits and *N. benthamiana* TOR by both RAPTOR1::PafA and LST8‐1::PafA constructs. The proteins specifically labeled by RAPTOR1::PafA and/or LST8‐1::PafA baits also included 21 of the 66 proteins previously reported as TORC subunit interactors (Figure S1C,D, Supporting Information), confirming that the PafA fusions incorporated into the TORC. Heterologous expression of the TORC PUP‐IT constructs in *N. benthamiana* thereby provides a strong and rapid platform to identify TORC interactors.

### 2.2 Sucrose Treatment Identifies Sugar‐Dependent and ‐Independent TOR Complex Interactions in Arabidopsis

TORC activities are cell type‐, tissue‐, and environment‐dependent.^[^ ^22^ ^]^ Previous efforts to characterize the TOR interactome focused largely on cell cultures and noted limited overlap with experiments from whole plants or seedlings.^[^ ^11^ ^]^ Although our results from heterologous expression of LST8‐1 and RAPTOR1 PUP‐IT baits in *N. benthamiana* were promising (Figure S1C,D, Supporting Information), the results were similarly limited to a single plant cell type—the infiltrated epidermal leaf cells. Additionally, the conservation of TORC between *Brassicaceae* and *Solanaceae* is not well defined, and might result in unexpected PPIs. To comprehensively map native PPIs in whole seedlings, we transformed our PUP‐IT constructs stably into Arabidopsis wild‐type plants. Given the prominent role of the TORC in sugar sensing and signaling, we assessed resource‐starved seedlings treated with either sucrose or mannitol (control). We first confirmed that sucrose treatments induced a TOR‐dependent growth response that could be blocked with the TOR‐inhibitor AZD8055 (Figure S2A,B, Supporting Information). Through label‐free mass spectrometry, we identified several hundred putative interactors for LST8‐1::PafA and RAPTOR1::PafA, including 40 proteins identified in StringDB and/or by Van Leene at al.^[^ ^11^ ^]^ (Figure 1B,C). However, we observed substantial discrepancies in identified proteins between both the sucrose and mannitol treatments as well as between the LST8‐1::PafA and RAPTOR1::PafA constructs, respectively. Indeed, we found 27% (100/367) common interactors between LST8‐1::PafA and RAPTOR1::PafA and only 7% (27/367) in both treatments (Figure S3A,B, Supporting Information). These discrepancies likely represent a combination of real changes in the TORC during the treatments and LST8‐1 and RAPTOR1 interactions occurring independently of TORC, but might also include a degree of background noise.

### 2.3 An Inducible PUP‐IT Approach to Identify Sugar‐Related Changes in the TORC Interactome

To increase sensitivity and improve signal‐to‐noise ratio of the FLAG::PUP(E) labeling, we next generated transgenics with constructs controlling FLAG::PUP(E) expression with a 𝛽‐estradiol inducible XVE cassette (Figure S1, Supporting Information). Here, we further confirmed PUP‐IT constructs were functional by complementing the RAPTOR1 loss‐of‐function phenotype (Figure S2C,D, Supporting Information). The XVE allowed us to induce labeling at a defined time point before harvesting with minimal promoter bleed‐though (Figure S4, Supporting Information). This reduced background labeling and possible side‐effects of excessive pupylation, thus allowing us to better target RAPTOR1 and LST8‐1 interactors when they were preferentially engaged with TORC. Using these lines, we induced 5 day‐old seedlings with β‐estradiol for 12 h and then supplemented the media with sucrose or mannitol for 4 or 24 h. Differential enrichment analysis of FLAG::PUP(E)‐tagged proteins yielded 906 putative TORC interactors across conditions and constructs, including 43 previously identified interactors (Figure 1D,E). Crucially, the inducible constructs showed better consistency between treatments and baits, finding 42% of putative interactors (384/906) in both baits and 45% (404/906) in both treatments (Figure 1F,G; and Figure S3C, Supporting Information). With this more stringent control over labeling times, we could also observe treatment‐specific roles of TORC interactors. Sugar‐starved conditions favored interactions related to protein translation, while sucrose treatment shifted the interactions toward carbon allocation and metabolism (Figure S3D,E, Supporting Information).

As indicated above, it is possible that some interactors, especially those identified by only one of the two baits, might be interacting with the bait mainly when it is not part of the TORC. To improve the likelihood of identifying true TORC interactors, we therefore also generated PafA fusions of the *Saccharomyces cerevisiae* 12‐kDa cytosolic FK506‐binding protein (*Sc*FKBP), which complexes with Arabidopsis TOR after binding rapamycin, impeding growth^[^ ^23^ ^]^ (Figure 1A; and Figure S1, Supporting Information). The rapamycin‐dependent TOR‐binding of the *UBIpro*:*Sc*FKBP::PafA fusion protein was confirmed by treating stably transformed wild‐type Arabidopsis lines with rapamycin, leading to an expected growth inhibition (Figure 1H; and Figure S5, Supporting Information). Notably, LST8‐1, RAPTOR1, and TOR were all highly enriched in the *Sc*FKBP bait (using a constitutively expressed FLAG::PUP(E)), confirming efficient and sterically flexible labeling of TORC interactors (Figure 1I). The three baits provided partly overlapping and partly complementary aspects of the TORC interactome. Depending on the bait, construct type, treatment and time point, the individual experiments identified between 65 and 499 proteins as putative TORC interactors (Table S3, Supporting Information), including up to 38 proteins previously reported to be TORC interactors, with various degrees of overlaps between bait proteins (Figure S6, Supporting Information).

### 2.4 Phosphoproteomics of Pupylated Proteins Pinpoints Putative Direct Substrates of TOR and Associated Kinases

**Figure 2:**
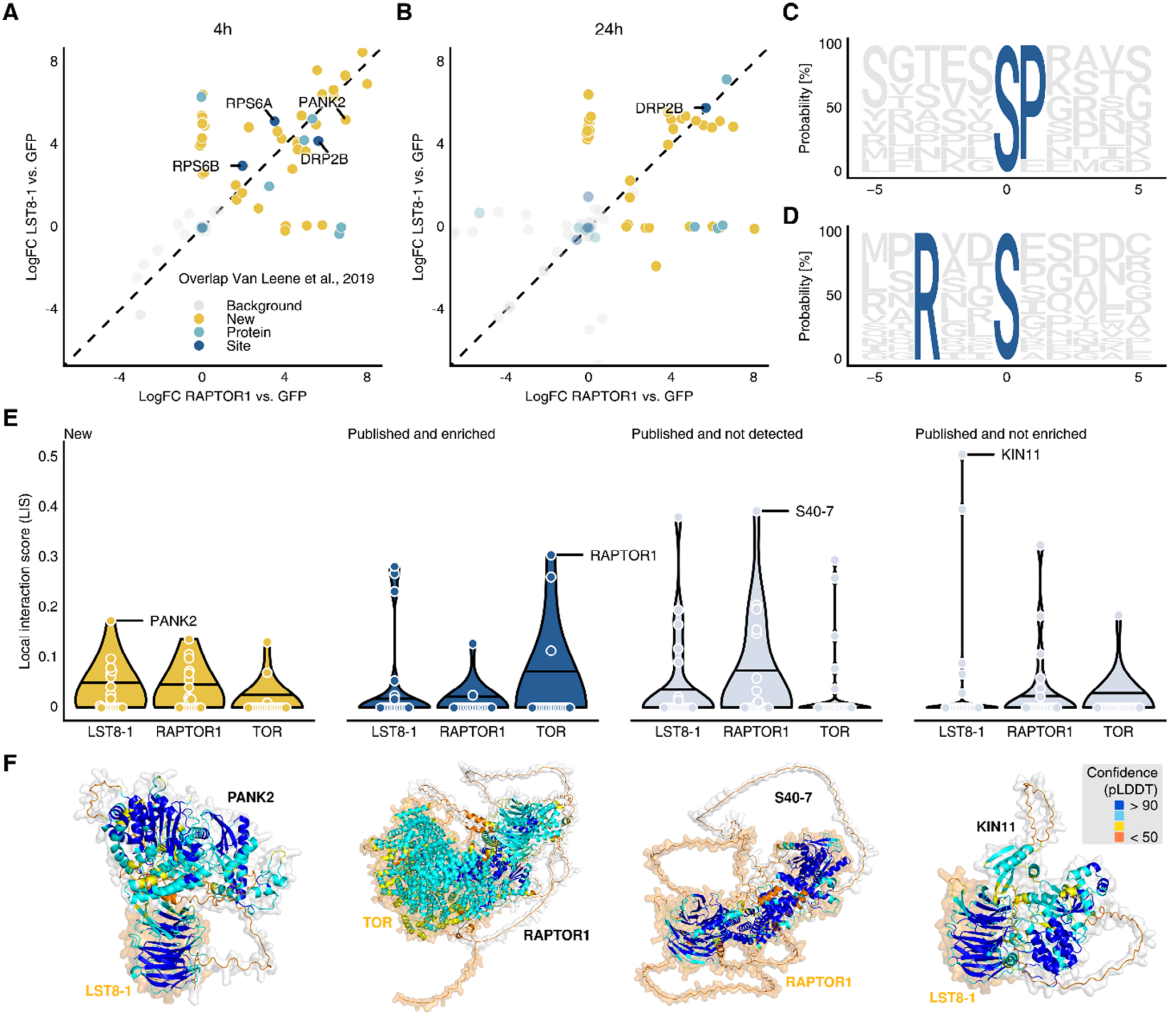
Identification of phosphorylated direct interactors of the TOR complex and in silico corroboration using AlphaFold2. A,B) Phosphorylated proteins enriched in LST8‐1 and RAPTOR1 against the GFP control after 4 h A) and 24 h B) of sucrose treatment and FLAG::PUP(E) induction. Phosphorylation sites previously associated with TOR are indicated in blue, new sites in proteins previously reported to be phosphorylated by TOR in turquoise. Fold changes are calculated from *n* = 3 replicates using MsqRob2, *p*‐values are corrected for multiple comparisons using the Benjamini–Hochberg FDR method. C,D) The two significantly enriched motifs among the identified phosphorylation sites. The “SP” motif C) mirrors previous reports from TOR substrates, while the “RxxS” motif D) has previously been associated with TOR downstream interactor S6K1. E) Interactions of 20 proteins from the four shown groups with the TORC components TOR, LST8‐1, and RAPTOR1 were predicted using AlphaFold2 multimer. Vertical lines indicate the median of the local interaction score (LIS) distribution by group. F) Predicted structures of the top‐scoring interactions for each group: newly identified TORC interactor PANK2, TORC subunit RAPTOR1, senescence regulator S40‐7, and KIN10 paralog KIN11. Models are colored by predicted local distance difference test (pLDDT), with values above 70 indicating high confidence predictions.

Beyond the characterization of TORC interactors, PUP‐IT also provides unique opportunities to identify direct TORC kinase substrates. Previous reports used the overlap between untargeted phosphoproteomics in combination with TOR inhibitors and independent TORC pull‐down experiments to find TORC kinase targets.^[^ ^11^ ^]^ By contrast, PUP‐IT allows us to directly enrich the pupylated protein lysates for phosphorylated peptides, generating the overlap between direct interactors and differentially phosphorylated sites from a single experiment. This approach, used on inducible FLAG::PUP(E) for the RAPTOR1 and LST8‐1 constructs, identified 79 unambiguously localized phosphosites enriched in TORC baits (**Figure** **2**A,B). Previously reported TORC phosphorylation targets were enriched in the early sucrose response using both LST8‐1 and RAPTOR1 baits (Figure 2A). These included ribosomal proteins RPS6A and RPS6B as well as dynamin‐related protein DRP2B. New putative TORC kinase substrates enriched more than 30‐fold in both LST8‐1 and RAPTOR1 baits included proteins associated with transcription/translation‐regulation (5/11), water/H+‐transport (4/11) as well as IQD32, and pantothenate kinase 2 (PANK2). These phosphorylation sites were enriched in sequences matching previously described TORC motifs. For example, the TOR‐associated motif “SP” and the “SxxP” motif associated with TOR‐downstream target S6K1 were 5.7‐ and 4.3‐fold enriched, respectively (Figure 2C,D). Of the known TORC interactors that we found phosphorylated in this analysis, RAPTOR1 carried the “SP” motif and RPS6B and DRP2B, identified as potential TORC kinase substrates by Van Leene et al.,^[^ ^11^ ^]^ carried the RxxS motif (Table S4, Supporting Information). The dual enrichment of proteins that were both FLAG::PUP(E)‐labeled and phosphorylated therefore provides an excellent tool to map phosphorylation dynamics throughout TOR‐mediated signaling, although the identified candidates will need to be unambiguously confirmed as direct TOR substrates in follow‐up studies.

### 2.5 In Silico Protein Modeling Supports Newly Identified TORC Interactors

PPIs have recently seen a surge in novel deep learning methods capable of accurately predicting the structures of multimeric protein complexes from their amino acid sequences.^[^ ^24^ ^]^ To further assess and benchmark our results, we, therefore, used AlphaFold‐Multimer to predict the likelihood of direct TORC PPIs with i) interactors newly identified in this study, ii) known interactors also detected using PUP‐IT, iii) published interactors not identified in our proteomics experiments, and iv) published interactors found but not enriched in our PUP‐IT data (Figure 2E,F). We found evidence for direct PPIs with a TORC component (TOR, LST8‐1, RAPTOR), defined as a local interaction score greater than zero, for proteins from all four groups (the structures of all predicted interactions are available from https://doi.org/10.5281/zenodo.14960064). However, the median support for TORC interactors identified for the first time in our work was higher than that of previously published interactors found in our data (Figure 2E). For instance, we discovered PANK2 (Q8L5Y9; Figure 2F), the enzyme catalyzing the rate‐determining step in CoA synthesis, which exhibited phosphorylation at S46 enriched in the TORC‐interacting fraction (Figure 2A), making it a promising candidate to link TOR signaling with CoA biosynthesis. This renders structure prediction‐based approaches as powerful tools for corroborating novel TORC interactors while providing tentative binding modes for further validation studies.

### 2.6 Integration of PUP‐IT Data Yields Finely Resolved Interactomes of the TOR Complex to Dissect Sugar Signaling

**Figure 3:**
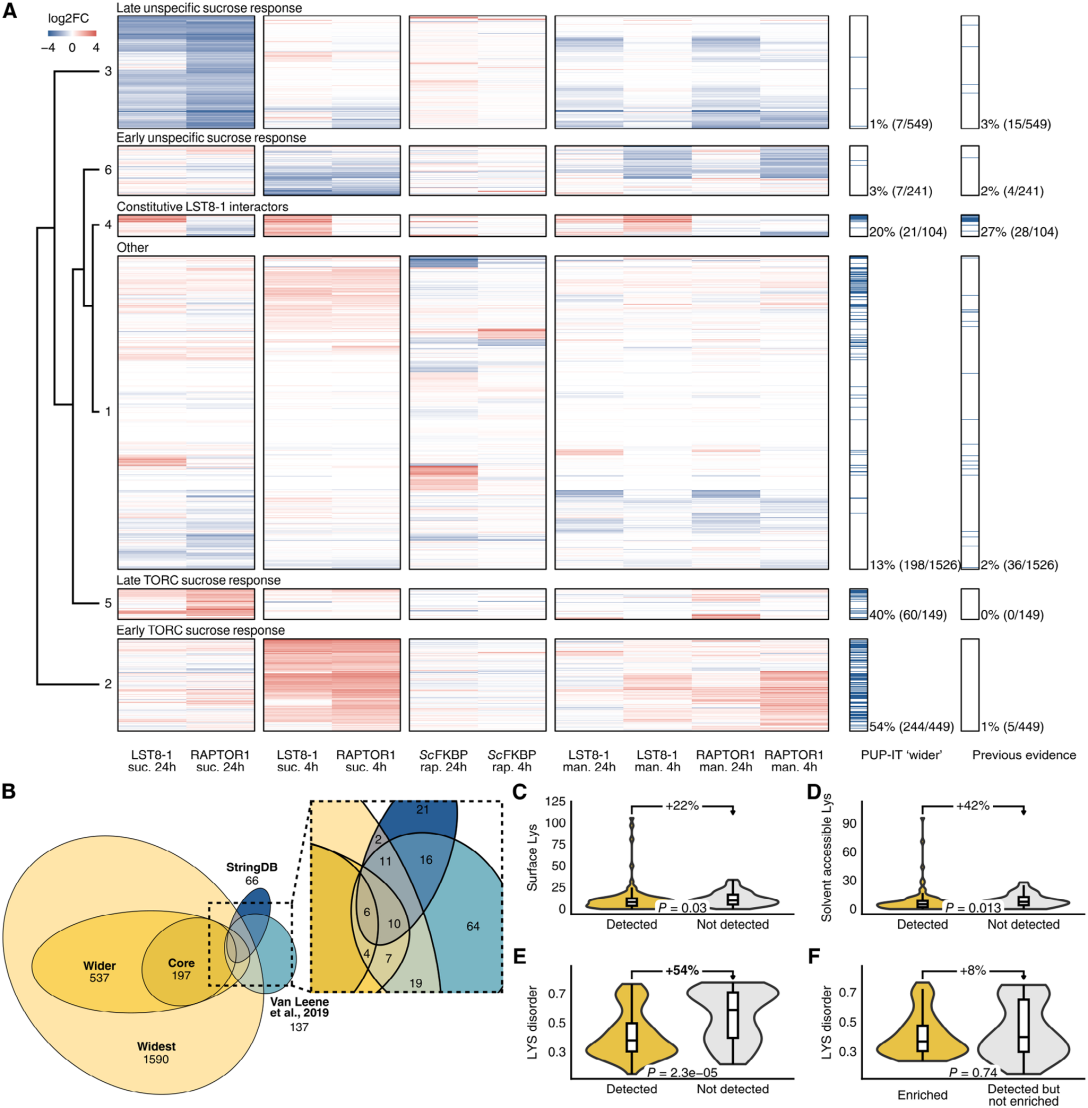
Integrating treatment‐ and bait‐specific data yields comprehensive and robust TOR interactomes. A) 2D clustering of treatments and all detected proteins based on protein enrichment in the used baits against the corresponding control (GFP for LST8‐1 and RAPTOR1, no rapamycin for *Sc*FKBP). When missing (i.e., undetected) in an experiment, proteins were imputed as unchanged (0). Annotations on the right side indicate whether each protein was classified as part of at least the “wider” interactome as defined here (panel B), and whether they have been identified as TORC interactors in previous reports. B) The three interactomes defined in this study and their overlap with TORC PPI data from StringDB and Van Leene et al.^[^ ^11^ ^]^ Numbers in the full Venn diagram represent total counts of each circle, while numbers in the zoomed inset represent those of specific overlaps. Note that the overlap between StringDB and Van Leene et al.^[^ ^11^ ^]^ is partially artificial, as the data from that paper is part of StringDB. C–F) Lys accessibility for pupylation in known TORC interactors detected (*n* = 88) and not detected (*n* = 70) in our data. C) Number of Lys residues at the protein surface (residues depth <3.6 Å). D) Number of solvent accessible (RSA > 0.7) Lys residues. E) Disorder in stretches surrounding solvent accessible Lys residues. F) Disorder in stretches surrounding solvent accessible Lys residues, compared between previously reported TORC interactors enriched (*n* = 59) or detected but not enriched (*n* = 29) in our data. *p*‐values are from two‐sided, Welch‐corrected *t*‐tests.

Proteomic analyses of proximity labeling approaches have an inherently stochastic nature that limits the conclusions one can draw from single experiments. To generate a robust and specific TORC interactome, PPI data from the five different constructs in Arabidopsis, i.e., LST8‐1 and RAPTOR1 with constitutive FLAG::PUP(E), LST8‐1, and RAPTOR1 with inducible FLAG::PUP(E) and *Sc*FKBP, needed to be integrated. Such integration allows differentiating between proteins that interact with LST8‐1, RAPTOR1, or ScFKBP when they are not part of TORC, and proteins that are enriched in multiple baits and experiments, supporting their interaction with TORC. We first categorized putative interactors according to their propensity to interact with different baits in a treatment‐ and time‐dependent manner (**Figure** **3**A). All proteins detected in our proteomics data sets were therefore clustered according to their log fold enrichment as compared with the control bait (GFP for RAPTOR1/LST8‐1) or control treatment (no rapamycin for *Sc*FKBP). This approach clustered the proteins into six distinct modules (Figure 3A). Modules 3 and 6 comprised proteins interacting with GFP, and are thus less relevant for this study. By contrast, modules 2, 4, and 5, and to a lesser extent 1, contained putative TORC bait interactors across multiple baits and treatments. Module 2 represented the early TORC sucrose response, with proteins specifically enriched in the 4 h time point of LST8‐1 and RAPTOR1 under sucrose treatment. Module 4 comprised constitutive interactors of LST8‐1, found at both time points independently of treatment. Module 5 represented late TORC sucrose response and constitutive RAPTOR1 interactors. Previously reported TORC interactors were over‐represented in module 4, i.e., 27% of the module had previous evidence compared with 3% in the module with the next highest proportion. On one hand, this makes proteins in module 4 promising candidates for further investigation. On the other hand, the tight clustering of known interactors into this module corroborates our hypothesis that previously performed pull‐down and TAP experiments were biased toward strong, constitutive interactors, missing the transient and treatment‐specific interactors comprising modules 2 and 5. Altogether, the hierarchical clustering provides biological context for each putative interaction, indicating which TORC member it is most closely associated with and under which conditions it occurs.

To define the experimental and biological robustness of detected interactions, we generated three proposed interactomes using increasingly stringent filtering thresholds (Figure 3B). Here, the widest interactome includes any proteins found enriched with at least two bait constructs. The wider interactome includes proteins enriched in at least three bait constructs that were never, in any possible between‐group comparison, found enriched in the GFP bait. Finally, proteins in the core interactome were found enriched in at least five bait constructs and never with GFP, or identified as putative kinase substrates of TOR (cf. Figure 2). To attempt a rough benchmarking of our proposed interactomes, previously reported interactions were categorized as high‐confidence, while those with proteins predicted to the thylakoid (and thus unlikely to directly interact with TOR) were designated low‐confidence; the ratio between those two categories can be considered a rough proxy for the experimental signal‐to‐noise ratio (**Table** **1**). Interactive visualizations of these interactomes are included as File S2 (Supporting Information), encoding the robustness of a given interaction as the cumulative sum of the observed log fold enrichment compared with the control. To estimate how the PUP‐IT‐based interactors compared to those previously determined, we visualized the overlap with data from StringDB and previous work by Van Leene et al.^[^ ^11^ ^]^ The corresponding Venn diagram shows that 42% of previously reported interactors are part of the widest PUP‐IT interactome (Figure 3B and Table 1). The overlaps of all three interactomes with previously published data was much larger than expected by chance (hypergeometric test, *p* < 0.00001). Indeed, our experiments supported 17/18 (94%) of the interactors detected in both cell cultures and seedlings by Van Leene et al.,^[^ ^11^ ^]^ confirming the capacity of our approach to identify high‐confidence interactors. When characterized by their metabolic roles through KEGG enrichment, known interactors (also found in our study) were enriched in TORC‐associated pathways such as vitamin B metabolism and autophagy, while interactors only found in this study were linked to, e.g., the TCA cycle (Figure S7, Supporting Information). Finally, to identify specific interactors of TORC, as opposed to hub proteins and other promiscuous interactors, we compared the three proposed interactomes with a database of PUP‐IT experiments using unrelated baits. This comparison suggested that roughly 80% of TORC interactors identified here—including 30 known interactors such as the prefoldin complex—also commonly interacted with proteins not involved in sugar signaling or carbon allocation (Figure S8, Supporting Information). The remaining 288 interactors included TORC components TOR, LST8‐1, and RAPTOR1, along with known interactors, such as THY1 and 2, CCT1 and 2, and eIF2B subunit delta. The additional, previously undescribed 279 interactors in this group provide a unique resource for the identification of novel TORC‐specific regulatory pathways (Files S2 and S3, Supporting Information).

**Table 1:** Benchmarking of different thresholding stringency when defining the TOR interactome based on PUP‐IT data.

Interactome	Min. sig. experiments	Stringent filter of promiscuous interactors	Total interactors	StringDB	^[^ ^11^ ^]^	Predicted to the thylakoid	High vs low confidence interactor ratio
Widest	2	FALSE	1591	29	57	54	1.1:1
Wider	3	TRUE	538	16	27	10	2.7:1
Core	5	TRUE	198	6	10	1	10:1

Finally, to gauge the extent to which our protein‐level interactome could be recreated using transcriptomic data, we created a coexpression network of high‐confidence candidates comprising the “core” interactome, putative kinase substrates, and the proteins forming cluster 4 (cf. Figure 2A). This analysis showed that while there was a higher degree of coexpression among the candidates identified with PUP‐IT than expected in a random group of genes (Figure S9A, Supporting Information), there was no further relationship between degree of coexpression and PUP‐IT enrichment (Figure S9B, Supporting Information). Nonetheless, community detection based on a network filtered to the 99th percentile of coexpression resulted in several clusters, which will be useful for further functional characterization of TORC interactors (Figure S9C, Supporting Information).

### 2.7 PUP‐IT Preferentially Targets Lysine Residues in Ordered Protein Regions

Besides potential false positives in previously published experiments and PPIs only occurring in conditions not covered by our experimental set‐up, prey‐specific decreases in PUP‐IT labeling efficiency might also contribute to the absence of some expected interactors from our data. Indeed, since PafA directly ligates PUP(E) to Lys residues, Lys accessibility and ligation‐susceptibility are prerequisites for efficient pupylation—at least theoretically.^[^ ^25^ ^]^ To test this, we analyzed the AlphaFold2‐predicted structures of all 158 previously reported physical interactors. Both detected (*n* = 88) and not detected (*n* = 70) published TORC interactors had numerous Lys residues that were close to the protein surface (residue depth < 3.6 Å; Figure 3C) and solvent accessible (relative solvent accessibility > 0.7; Figure 3D). Curiously, not detected proteins had higher numbers of accessible Lys residues than detected proteins. Predicted disorder of the regions containing accessible Lys, however, was considerably (54% median difference) higher in undetected versus detected proteins (Figure 3E). The overall bias in Lys disorder disappeared when comparing the enriched (i.e., part of at least the “wider” interactome; *n* = 59) to the detected, but not enriched proteins (*n* = 29; Figure 3F). Finally, we excluded the alternative explanation of a disorder‐dependent bias in tryptic digestion by comparing relative peptide intensity to cleavage site disorder in the analyzed proteins, showing no relationship (Figure S10, Supporting Information). Together, these observations suggest that pupylation of a protein requires accessible, ordered Lys residues.

## 3 Discussion

Nearly all protein functions are modulated through protein–protein interactions. This is especially true for TORC, which forms an interaction hub that directs a multitude of different pathways in growth and development throughout the plant's life cycle. While intense research has been undertaken on TOR in mammals and yeast, our knowledge of interactions with the single TORC complex found in plants remains fractional. Candidate‐driven work has identified several important binary interactions. As a general theme, the recent discovery of CIPK–TORC interaction reinforced a model in which proteins of the SnRK superfamily generally suppress TORC, shifting the growth‐defense trade‐off toward the defense.^[^ ^26^ ^]^ However, given the multitude of its regulatory roles, most of TORC interactors are likely still unknown. The TORC protein interactome was previously tackled in a landmark study using affinity purification assays.^[^ ^11^ ^]^ Despite their thorough experimental design, the number of identified interactors robustly identified in whole seedlings and multiple baits was limited (Figure S11, Supporting Information). This may partly be due to the inherent limitations of affinity purification approaches, specifically regarding transient PPIs or complexes of limited stability.^[^ ^27^ ^]^

More recently, proximity labeling has been successfully adapted for use in plant systems, generating valuable resources for the characterization of PPIs that would typically elude affinity purification‐based approaches.^[^ ^28^ ^]^ Here, we captured the TORC interactome using PUP‐IT; a fully genetically‐encoded proximity labeling approach. On the one hand, the rapid pupylation reaction should ensure labeling of constitutive and transient interactors, as also attempted in protoplasts,^[^ ^29^ ^]^ sharing the primary advantage of other proximity labeling techniques, such as TurboID. On the other hand, the pupylation reaction requires physical contact of the PafA ligase with the substrate, avoiding the primary drawback of TurboID: unspecific labeling by freely diffusing activated biotin tags.^[^ ^30^ ^]^ We combined this technique with multiple TORC subunits as baits to characterize TORC from different angles and to provide a valuable resource for the TORC community. Given that there is no “gold standard” for the accuracy of the TORC interactome in plants, one of the few tangible measures of success is the overlap in identified interactors when using different TORC components as baits. Indeed, our PUP‐IT results for LST8‐1 and RAPTOR1 exhibit very similar levels of overlap to affinity purification‐based results, even though we identified roughly ten times more putative TORC interactors (Figure S11, Supporting Information). Recently, Persyn et al.^[^ ^18^ ^]^ published a comprehensive mapping of the Nitrogen‐dependent signaling cascade downstream of TOR, using affinity purification and biotin‐based proximity labeling. A comparison with these results shows that our PUP‐IT based approach achieved a larger overlap between baits, identified and higher number of total interactors and recovered a larger number of known interactors^[^ ^18^ ^]^ (Figure S8 and File S3, Supporting Information). Further enriching our pupylated proteins for phosphorylated peptides allowed us to identify putative direct TOR kinase substrates. Although we cannot fully avoid proteins phosphorylated prior or after their interaction with TOR, our approach represents a significant improvement in candidate confidence by providing evidence for direct interaction and phosphorylation from a combined experiment.

The novel interactors identified here include candidates that may link TORC to various aspects of carbon allocation and metabolism. Interactors that, like PANK2, were supported by both PUP‐IT and Alphafold2 include for example UXS1 (Q8VZC0), HDT2 (Q56WH4), and FBA8 (Q9LF98), which represent possible links of TORC activity with cell wall biosynthesis, histone acylation, and gluconeogenesis, respectively. On the other hand, the results include numerous almost entirely uncharacterized proteins, which might lead to new aspects of TORC signaling. We also provide data on sugar‐dependent interactions, which might provide new directions for investigating TORC‐dependent source‐sink dynamics.^[^ ^31^ ^]^ The resources presented herein should thus establish stepping stones for future exciting genetic, biochemical, and structural investigations to fully characterize these interactions.

Despite the advances presented here, results from large‐scale proximity‐labeling or pull‐down experiments remain rough approximations of real interactomes. Stochastic errors and undetected systemic biases in sample preparation or MS analysis likely account for the absence of some expected proteins from our data. Indeed, several well‐established interactors of TORC components, including KIN10, PIN2, S6K1, and TAP46, were detected neither in our experiments nor in previous reports.^[^ ^11^ ^]^ Others, such as ROP2 and EIN2, both established TORC interactors missed by published pull‐down or TAP experiments, were enriched with our LST8‐1 and *Sc*FKBP baits, respectively. However, being only discovered with a single bait, they did not clear the stringent filtering thresholds to be included in the proposed interactomes. Some of the remaining “missingness” in our data, including most of the well‐known TORC interactors, is likely due to PUP‐IT‐specific sensitivity to high levels of disorder in surface accessible Lys (Figure 3E): S6K1, among many other examples, has a median Lys disorder of 0.67. Indeed, such preference for ordered proteins has previously been shown for the functionally analogous process of ubiquitination.^[^ ^32^, ^33^, ^34^ ^]^ Others, such as KIN10, are relatively ordered (median Lys disorder 0.361), suggesting other reasons for their absence from our and previous data. Surprisingly, close KIN10 paralog KIN11 (80.4% identity) was found in our data, but not enriched with TORC baits either, suggesting that the absence of SnRK1 subunits from our interactomes might have biological, not technical, causes. Given the size of TORC—the fully resolved human mTORC1 is almost 30 nm in diameter^[^ ^35^ ^]^—steric hindrance might also preclude the PafA‐tagged baits to label all TORC interactors. Indeed, the LST8‐1 bait did not significantly enrich RAPTOR1 protein or vice versa, supporting the supposition of steric separation between the two. Relatedly, RAPTOR2, and LST8‐2, the close paralogs of RAPTOR1 and LST8‐1, respectively, are likely mutually exclusive with their paralogs within a given TOR complex, precluding detection with RAPTOR1/LST8‐1 baits. Additionally, RAPTOR2 has around 70% lower expression levels than RAPTOR1 in seedlings, while LST8‐2 has low to undetectable expression levels in Arabidopsis.^[^ ^21^ ^]^ Finally, despite our efforts to minimize false positives with various controls and filtering steps (Table 1), they can not be avoided entirely. Of the 487 annotated proteins forming the “Wider” interactome, 30 (6%) appear to reside exclusively in ER lumen, mitochondria or plastids. However, previously reported interactors were also enriched in, e.g., ER lumen proteins (Figure S7, Supporting Information), RAPTOR1 has been reported to localize to mitochondria,^[^ ^36^ ^]^ and the chloroplastic protein RSH3 was recently shown to directly interact with TORC.^[^ ^37^ ^]^ Given the mounting evidence for unexpected TORC interactions, we hesitate to exclude these proteins from our proposed interactomes.

The discussed limitations notwithstanding, our data elucidate the TORC interactome in unprecedented detail and provides a rich resource to untangle the functions of TORC in plant biology. The raw data, proposed interactomes, used vectors and transgenic seeds are all available to the community and should provide a stepping‐stone for future insights into the mechanism behind the numerous roles of TORC in plants.

## 4 Experimental Section

### Constructs

Gibson assembly (NEBuilder HiFi DNA assembly mix, New England Biolabs) and Gateway cloning (Gateway LR Clonase II Enzyme mix, Invitrogen) were employed to generate all the constructs in this manuscript. The primers used for fragment amplification are listed in Table S1 (Supporting Information).

To create the PUP‐IT entry clones, PCR amplified CDS fragments of RAPTOR1, LST8‐1, and of *Sc*FKBP, driven by the promoter of Arabidopsis Ubiquitin10, were subcloned into a TOPO entry vector. The entry clones were subsequently LR cloned to create destination vectors with FLAG::PUP(E) expressed under a constitutive (pMDC32) or a β‐estradiol inducible (pFM) 35S promoter. Sequences of all vectors are available in File S1 (Supporting Information). For plant transformation, destination clones were transformed into the Agrobacterium strain GV3101; the pSoup helper plasmid was added where necessary.

### Plant Material and Growth Conditions

*N. benthamiana*, and *Arabidopsis thaliana* lines Col‐0 and *raptor1* (SALK_101 990) were used as the hosts for protein expression. For the plant transformation and seed propagation, *N. benthamiana* and Arabidopsis seedlings were transferred to soil pots in a greenhouse under set conditions (16 h light/21 °C and 8 h dark/19 °C). Before that, Arabidopsis seedlings were grown in a growth cabinet under long‐day conditions (16 h light/21 °C and 8 h dark/19 °C) on half Murashige and Skoog (MS) plates with 1% sucrose.

Agrobacterium strain GV3101 carrying the respective destination clones were cultured and co‐infiltrated into *N. benthamiana* leaves with RNA silencing suppressor p19 (OD600 = 0.5 in infiltration buffer) as previously described.^[^ ^38^ ^]^ For pMDC32 samples, the tissues were collected 2 days after infiltrated plants growing in the greenhouse. For pFM samples, 100 µm β‐estradiol was painted on the leaf surface a day after the infiltration. Then tissues were collected after the β‐estradiol induction for another day. Total proteins were extracted with RIPA buffer (123 mm Tris, 750 mm NaCl, 5% Igepal, 5% sodium deoxycholate, 0.5% SDS) to detect the FLAG‐tagged proteins by western blot with FLAG antibody (Merck, F3165, diluted 1:2000).

Stable transgenic Arabidopsis carrying pMDC32 or pFM derived vectors were generated through floral dipping as previously described.^[^ ^39^ ^]^ Hygromycin selection on ½ MS plates was applied to the lines carrying pMDC32, while BASTA selection in soil was applied to the lines carrying pFM. For the positive transgenic plants, the T2 generations from pMDC32 or pFM were grown on ½ MS plates with 1% sucrose supplied with Hygromycin or BASTA and 20 µm β‐estradiol respectively. Then total proteins were extracted from 10‐days old seedlings with RIPA buffer to detect the FLAG‐tagged proteins by western blot with FLAG antibody.

To generate materials for proteomics, *N. benthamiana* tissues were prepared as described above. *Arabidopsis* transgenic stable and inducible lines were sterilized with 3% bleach solution, stratified at 4 °C for 3 days. The seedlings were grown in a growth cabinet with 4 h light/21 °C and 20 h dark/19 °C in liquid ½ MS media with a ratio of 600 seeds/100 mL. The culture bottles were placed on a shaker at 120 rpm.

The pMDC32 Raptor::PafA, pMDC32 LST8::PafA, and pMDC32 GFP::PafA seedlings were supplemented with 90 mm sucrose or mannitol and placed in the dark for 24 h after 5 days of growth in the conditions mentioned above (4 h light/20 h dark).

The pMDC32 *Sc*FKBP:PafA seedlings were grown for 5 days (4 h light/20 h dark) and then supplemented with 90 mm sucrose with and without 10 µm rapamycin and placed in the dark after treatment. Seedlings were collected 4 and 24 h after supplementation.

The pFM inducible transgenic seedlings were induced by adding 10 µm β‐estradiol and placed in the dark after 5 days of growth (4 h light/20 h dark). 12 h after induction, 90 mm sucrose or mannitol was supplemented. Seedlings were collected 4 and 24 h after supplementation.

### Protein Extraction and Enrichment

Tissues from 3 to 4 *N. benthamiana* leaves (2–3 g) were sampled and ground in liquid nitrogen to a fine powder. Total proteins were extracted with extraction buffer (1:3 [W/V]‐ material: buffer), [20 mm Tris‐HCl, pH 7.4, 150 mm NaCl, 5 mm MgCl2, 1 mm EDTA, 1% Triton X‐100, and 0.1% protease inhibitor cocktail (Roche)] for 30 min on ice, and centrifuged at 4 °C at 17210 g for 30 min. The supernatant was divided into three equal amounts for the following independent immunoprecipitation triplicates, then incubated each with 100 µL equilibrated anti‐Flag antibody coupled magnetic beads (Thermo Scientific, A36797) for 2 h at 4 °C. Beads were washed three times with extraction buffer [20 mm Tris‐HCl, pH 7.4, 150 mm NaCl, 5 mm MgCl2, 1 mm EDTA, 1% Triton X‐100, and 0.1% protease inhibitor cocktail (Roche)], then washed three times with wash buffer [20 mm Tris‐HCl, pH 7.4, 150 mm NaCl, 5 mm MgCl2, 1 mm EDTA, and 0.1% protease inhibitor cocktail (Roche)] and three times with 1 X PBS. After that the beads were stored at –70 °C until LC‐MS/MS analysis.

A similar process was performed on tissues from Arabidopsis seedlings (1–2 g) with four independent transgenic lines mixed. Tissue was ground in a cold mortar with extraction buffer (1:3 [W/V] – material:buffer), [50 mm HEPES pH 7.5, 150 mm NaCl, 10 mm NaF, 5 mm EDTA, 1% Triton X‐100, 1% PVP40, 0.1% protease inhibitor cocktail (Roche), and 0.25% phosphatase inhibitor cocktail (Roche)]. Extraction was carried out for 1 h on ice and samples were then centrifuged at 17 210 g and 4 °C for 30 min. The supernatant was divided into three equal amounts for the following independent immunoprecipitation triplicates, then incubated each with 100 µL equilibrated anti‐Flag antibody coupled magnetic beads. Beads were washed three times with extraction buffer, then three times with wash buffer [50 mm HEPES pH 7.5, 150 mm NaCl, 10 mm NaF, 5m EDTA, 0.1% protease inhibitor cocktail (Roche), and 0.25% phosphatase inhibitor cocktail (Roche)] and finally washed three times with 1 X PBS buffer. The last wash was removed and the beads were stored at −70 °C until LC‐MS/MS analysis.

### Protein Digestion and Mass Spectrometry

Washed beads were incubated for 30 min with elution buffer 1 (2 m Urea, 50 mm Tris‐HCl pH 7.5, 2 mm DTT, 20 µg mL^−1^ trypsin) followed by a second elution for 5 min with elution buffer 2 (2 m Urea, 50 mm Tris‐HCl pH 7.5, 10 mm Chloroacetamide). Both eluates were combined and further incubated at room temperature overnight. Tryptic peptide mixtures were acidified to 1% TFA and loaded on Evotips (Evosep). Peptides were separated on 15 cm, 150 µm ID columns packed with C18 beads (1.9 µm) (Pepsep) on an Evosep ONE HPLC applying the “30 samples per day” method, and injected via a CaptiveSpray source and ten µm emitter into a timsTOF pro mass spectrometer (Bruker) ran in PASEF mode.

For phosphoproteomics, tryptic peptide mixtures were acidified to 1% TFA and purified with an EasyPep Peptide Clean‐up Plate (Thermo). Eluates were vacuum centrifuged to near dryness, resuspended in 20 µL Buffer A* (2% ACN, 0,1% TFA), and 5 µL loaded onto Evotips as Input. The remaining material was combined with 200 µL binding buffer (0.1 m glycolic acid, 80% ACN, 5% TFA), and incubated with 20 µL of washed and equilibrated 1:1 mix of Ti‐IMAC and Zr‐IMAC beads (20 mg mL^−1^, Resyn Biosciences) for 20 min, medium speed, using a Thermo KingFisher Purification System. Beads were washed once with 500 µL binding buffer, once with 500 µL wash buffer 1 (80% ACN, 1% TFA), and once with 500 µL wash buffer 2 (10% ACN, 0.2% TFA), for 2 min at fast speed. Phosphopeptides were eluted with 100 µL 1% ammonia for 10 min at medium speed, and the eluate acidified by addition of 10 µL 10% formic acid. Peptides were vacuum centrifuged to near dryness, resuspended in Buffer A*, and loaded onto EvoTips. Phospho‐enriched peptides were separated on a Pepsep 15 cm, 150 µm ID column packed with C18 beads (1.5 µm) using an Evosep ONE HPLC system applying the default 30‐SPD (30 samples per day) method. Column temperature was maintained at 50 °C. Upon elution, peptides injected via a CaptiveSpray source and 20 µm emitter into a timsTOF pro2 mass spectrometer (Bruker) operated in PASEF mode.

### Peptide Search

Raw data from the timsTOF‐PASEF runs was analyzed in FragPipe (v 21.1) as described elsewhere.^[^ ^40^ ^]^ Briefly, peptide spectrum matches (PSMs) were identified using MSFragger (v 4.0) with a precursor mass tolerance of 50 PPM, a fragment mass tolerance of 20 PPM, isotope errors of 0/1/2 and enabled mass calibration and parameter optimization. The Arabidopsis reference proteome UP000006548 and the *N. benthamiana* NbDE maximum coverage proteome^[^ ^41^ ^]^—both with added common contaminants and reversed decoy sequences—were used as databases for experiments in Arabidopsis and *N. benthamiana* respectively. Semi‐enzymatic in silico protein digestion was performed, allowing one or both peptide termini at a trypsin cleavage site, up to two missed cleavage sites, and a final peptide length of 7–50 amino acids or 500–5000 Da. M oxidation (+15.9949 Da), *N*‐terminal acetylation (+42.0106 Da), the GGE PUP(E) remnant at K residues (+243.0 Da) and, for phosphoproteomics, STY phosphorylation (+79.96 633 Da) were set as variable modifications; carbamidomethylation of Cys (+57.02 146 Da) was set as a fixed modification. PSM identification was rescored with MSBooster.^[^ ^42^ ^]^ PeptideProphet and ProteinProphet through Philosopher (v 5.1.0).^[^ ^43^ ^]^ were used to filter PSMs and peptides to 1% FDR. Phosphorylation localization was rescored with PTMProphet.^[^ ^44^ ^]^ Quantification was performed using the MaxLFQ method as implemented in IonQuant (version 1.10.12) with match between runs at a FDR of 1%—“MBR top runs” was adjusted to restrict matching to samples with the same bait.

### Differential Enrichment Analysis

Peptide quantification data from FragPipe was imported into R (v 4.3) and analyzed with MSqRob2 (v 1.10.0).^[^ ^45^ ^]^ Contaminant and decoy peptides were filtered out, the MaxLFQ intensities were log2 transformed and median‐centered. Peptides only detected in a single sample (within each MS run) and peptides from proteins only identified by a single ion (across MS runs) were filtered out. Missing values were categorized as missing‐at‐random (MAR; i.e., missing for technical reasons) with only one missing value per triplicate; otherwise as missing‐not‐at‐random (MNAR; i.e., missing for biological reasons). MAR values were imputed as 95% of the lowest observed value for the respective peptide by triplicate—accounting for the slight correlation between missingness and abundance even well above the detection limit.^[^ ^46^ ^]^ MNAR values were imputed using a MinProb approach, drawing from a distribution with a mean at the 0.5% quantile of observed intensity values by run and a standard deviation of 0.05. Aggregation to protein level, protein quantification and differential enrichment statistics were performed as implemented in MSqRob2.^[^ ^45^ ^]^ Arabidopsis homologs of *N. benthamiana* proteins were identified using MMseqs2.^[^ ^47^ ^]^ Proteins were annotated with Gene Ontology, Interpro, and ENSEMBL data using biomartr (v 1.0.7).^[^ ^48^ ^]^

### Network Analysis

Previously reported interactors of TORC members TOR (Q9FR53), LST8‐1 (Q9LV27), and RAPTOR1 (Q93YQ1) were downloaded through the StringDB API (https://string‐db.org v 12.0) and taken from the Supporting Information of previous publications.^[^ ^11^ ^]^ StringDB data were only included when the experimental evidence was at least moderate (>0.25). Protein levels were compared in all combinations of samples that only varied in one of bait, treatment, or time point. No comparisons across different mass spectrometry runs were made. The networks were filtered based on three constraints: number of biological replicates, unspecific enrichment and treatment‐dependency. A biological replication in this specific context was defined as an independent sample finding a given interaction. For example, if an interactor was enriched in experiment 1 with both LST8‐1 and RAPTOR1 baits and in experiment 2 with the LST8‐1 bait, this TORC interactor would be classified as thrice replicated. The unspecific enrichment filter, when applied, filtered out proteins that were also found enriched in the GFP bait in any of the tested comparisons. The treatment filter, when applied, filtered out interactors enriched in control conditions (mannitol/no rapamycin). Network visualization was done using the visNetwork R package (v 2.1.2).

### Analysis of Lysine Accessibility

To test whether the general accessibility of lysine residues might influence efficient labeling through PUP‐IT, all proteins reported as TORC interactors in StringDB or previous systematic analyses^[^ ^11^ ^]^ were grouped into those detected and/or enriched in the dataset and those that were not. Using AlphaFold2 models, lysine distance from the protein surface was estimated using DEPTH^[^ ^49^ ^]^ and relative solvent accessibility was estimated according to Tien et al.^[^ ^50^ ^]^ The disorder in 25 amino acid‐wide windows around easily accessible lysines (relative solvent accessibility > 0.7) was estimated from AlphaFold2 models as described by Piovesan, Monzon, and Tosatto,^[^ ^51^ ^]^ modified to include accessible surface area normalization according to Tien et al.^[^ ^50^ ^]^

### In Silico Screening of Protein–Protein Interactions

AlphaFold2‐Multimer (v2.3.1) was used to predict complexes for 80 candidate proteins in combination with 3 potential interactors. For each prediction, a single multimer model was generated, with one prediction per complex. Details on the AlphaFold2 configuration and the specific database versions used are available in Table S2 (Supporting Information). To classify interactions as positive or negative, the AlphaFold‐Multimer Local Interaction Score (AFM‐LIS;^[^ ^52^ ^]^) was applied with default parameters (commit hash: 5573a3b). Interactions were considered strong positives, if the LIS score was ≥ 0.203 and the Local Interaction Area (LIA) was ≥ 3432, interactions with LIS scores ≥ 0.073 and an average LIA ≥ 1610 were considered positive.

### Coexpression Analysis

Gene coexpression between proteins of interest were determined based on a published gene coexpression network^[^ ^53^ ^]^ constructed using Arabidopsis RNA‐sequencing (RNA‐Seq) samples from European Nucleotide Archive (ENA;^[^ ^54^ ^]^) based on the Two‐Tier Ensemble Aggregation (TEA‐GCN) method.^[^ ^53^ ^]^ Since gene coexpression strengths, in the form of Mutual Ranks,^[^ ^55^ ^]^ from the downloaded network were determined from different correlation metrics and in multiple subsets of RNA‐seq samples,^[^ ^53^ ^]^ the gene coexpression strengths were standardized to unit variance across the whole network before analysis to enhance interpretability.

### Data Analysis and Statistics

All general data analysis and plotting was performed in R (v 4.3) relying primarily on the tidyverse packages.^[^ ^7^ ^]^ Area‐proportional Venn diagrams were generated with the eulerr package (v 7.0.1).^[^ ^56^ ^]^ Modules of proteins and samples were calculated by hierarchical clustering (hclust; ward.D2 method; stats v 4.3); proteins not detected in a given MS run were imputed as unchanged (i.e., 0). For details about the statistical analyses during proteomics data analyses, see above sections. For all other experiments, sample sizes and used statistical tests are described in the figure legends. T‐, Tukey‐HSD, Dunn's, and Kruskal–Wallis tests were performed using the R packages stats (v 4.3) and dunn.test (v 1.3.5) with 𝛼 = 0.05. Phosphorylation motifs were detected in 11 amino acid windows using MoDL through MoMo.^[^ ^57^ ^]^

### Accession Numbers

CCT1, P28769/AT3G20050; CCT2, Q940P8/AT5G20890; DRP2B, Q9LQ55/AT1G59610; eIF2B subunit delta, Q9FFV8/AT5G38640; EIN2, Q9S814/AT5G03280; FBA8, Q9LF98/AT3G52930; HDT2, Q56WH4/AT5G22650; KIN10, Q38997/AT3G01090; KIN11, P92958/AT3G29160; LST8‐1, Q9LV27/AT3G18140; PANK2, Q8L5Y9/AT4G32180; PIN2, Q9LU77/AT5G57090; RAPTOR1, Q93YQ1/AT3G08850; ROP2, Q38919/AT1G20090; RPS6A, O48549/AT4G31700; RPS6B, P51430/AT5G10360; S40‐7, Q9LKA1/AT3G15040; S6K1, P42818/AT3G08730; *Sc*FKBP, P20081; TAP46, Q8LDQ4/AT5G53000; THY1, Q05762/AT2G16370; THY2, Q05763/AT4G34570; TOR, Q9FR53/AT1G50030; UXS1, Q8VZC0/AT3G53520.

## Conflict of Interest

The authors declare no conflict of interest.

## Author Contributions

S.Z., L.B., and D.P. contributed equally and also co‐first authors. S.P. and S.Z. designed the experiments. S.Z. generated the constructs in this manuscript, tested the TOR‐related PUP‐IT vectors in the *N. benthamiana* system via Western and proteomics, performed Arabidopsis transformation with agrobacterium carrying the pMDC and pFM vectors, selected out the Arabidopsis transgenic lines transformed with pMDC vectors and produced their proteomics data under conditions with sugar promoting seedling growth determined by AZD inhibition. B.Ö. tested the Rapamycin treatment for the *Sc*FKBP PUP‐IT expressed transgenic lines. D.P. and S.Z. selected out the Arabidopsis transgenic lines transformed with the pFM vectors. D.P. performed the proteomics with transgenic plants expressing the *Sc*FKBP PUP‐IT and the inducible PUP‐IT under different conditions. L.R., H.D., and A.S.H. performed the in silico PPI prediction. P.K.L., Q.W.T., and M.M. performed the coexpression analysis. L.B. analyzed all data, quantified Lys disorder, generated the figures, and wrote the manuscript. S.P., S.Z., and D.P. contributed to the writing.

## Supporting information



Supporting Information

## Data Availability

Raw proteomics data is deposited at MassIVE (https://massive.ucsd.edu), identifiers MSV000096657, MSV000096513, MSV000096862, MSV000096864 and MSV000096866. All analyzed data, used primers and resulting vector maps are compiled in the supplementary data files. The scripts for the complete proteomics and PPI‐network analyses in study, together with source data to run them, are available at https://doi.org/10.5281/zenodo.14960064. Plasmids and seeds are available directly from the authors upon request.
