# Supplementary material for: Pupylation‐Based Proximity Labeling Unravels a Comprehensive Protein and Phosphoprotein Interactome of the Arabidopsis TOR Complex
Source: Adv Sci (Weinh). 2025 Mar 24;12(19):2414496. doi: 10.1002/advs.202414496 (PMC12097154; doi:10.1002/advs.202414496)
Supplement: Supplementary file 1 — Supporting Information [file ADVS-12-2414496-s001.docx]

# **Supplementary material**

**Pupylation-based proximity labeling unravel a comprehensive protein and phosphoprotein interactome of the Arabidopsis TOR complex**

Shuai Zheng^1†^, Leonard Blaschek^1†^, Delphine Pottier^1†^, Luuk Robin Hoegen Dijkhof^2^, Beyza Özmen^1^, Peng Ken Lim^3^, Qiao

Wen Tan^3^, Marek Mutwil^3^, Alexander Sebastian Hauser^2^, Staffan Persson^1,4*^

1. Copenhagen Plant Science Center (CPSC), Department of Plant & Environmental Sciences, University of Copenhagen, Frederiksberg C, 1871, Denmark
2. Department of Drug Design and Pharmacology, Faculty of Health and Medical Sciences, University of Copenhagen, Copenhagen, 2100, Denmark
3. School of Biological Sciences, Nanyang Technological University, 637551, Singapore
4. Joint International Research Laboratory of Metabolic & Developmental Sciences, State Key Laboratory of Hybrid Rice,

SJTU-University of Adelaide Joint Centre for Agriculture and Health, School of Life Sciences and Biotechnology, Shanghai Jiao

Tong University, Shanghai, China

†These authors contributed equally to this work.

* staffan.persson@plen.ku.dk

Content

This document contains 11 supplementary figures and 3 supplementary tables on 16 pages.

## **Supplementary figures**


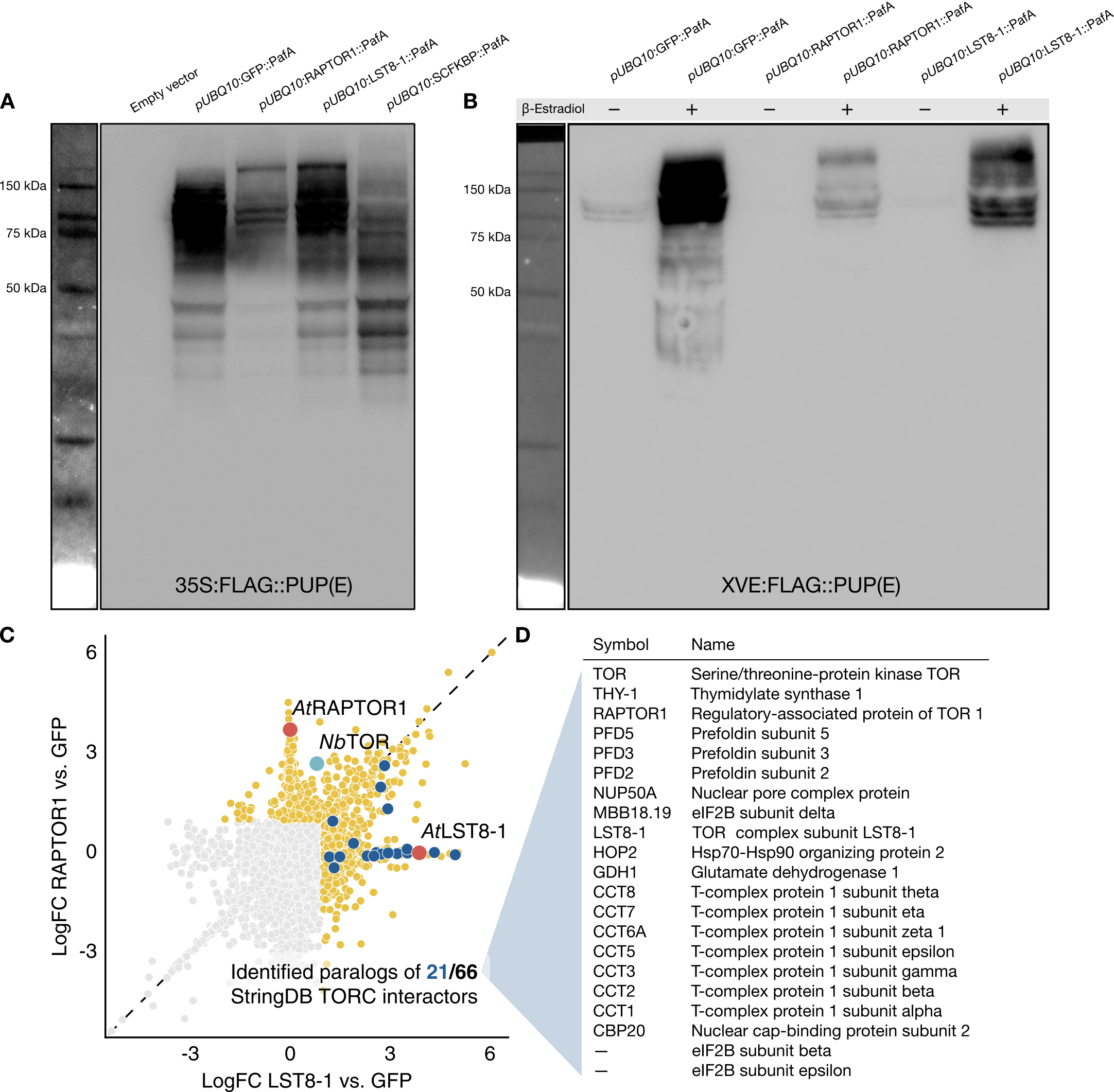


**Figure S1.** Transient PUP-IT in *N. benthamiana* leaves. **A,** Anti-FLAG Western blots of constitutive PUP-IT constructs expressed transiently in *N. benthamiana* leaves. **B,** Anti-FLAG Western blots of inducible PUP-IT constructs expressed transiently in *N. benthamiana* leaves. **C,** Proteins identified as interactors using LST8-1 and RAPTOR1 baits in *N. benthamiana* leaves. Yellow dots are significant interactors in at least one bait (log2FC >1 and P < 0.05). Recovered known interactors are indicated in blue, TOR in turquoise and the baits in red. Fold changes are calculated from *n* = 3 replicates using MsqRob2, *P*-values are corrected for multiple comparisons using the Benjamini-Hochberg FDR method. **D,** List of known TORC (i.e. TOR, RAPTOR1 or LST8-1) interactors that were significantly enriched in our experiment, corresponding to the blue dots in panel B.


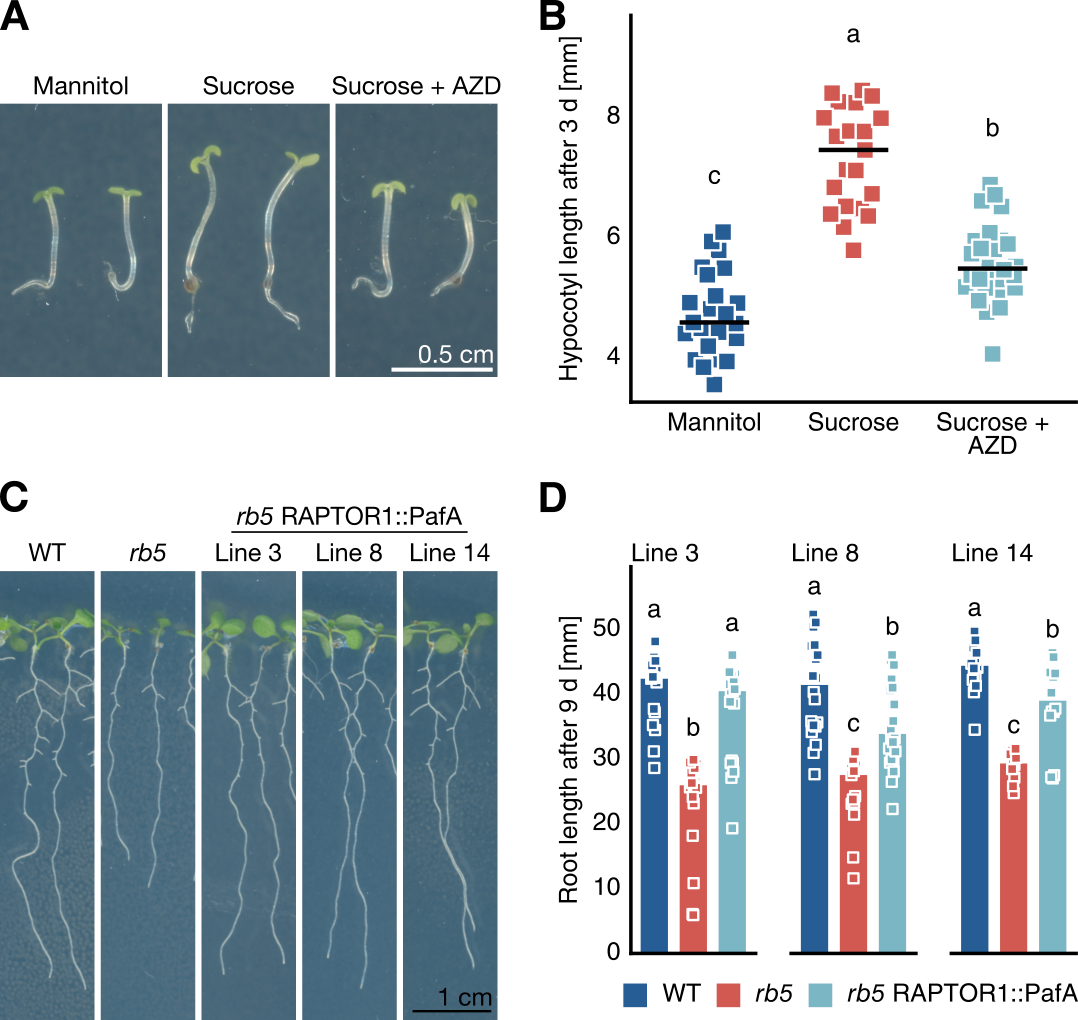


**Figure S2.** PUP-IT constructs are functional and sucrose treatments induce TOR-dependent growth responses. **A,** Seedlings grown for 5 d with 4h light and 20h dark in liquid medium, then supplemented with mannitol, sucrose, or sucrose and the TOR inhibitor AZD8055 (AZD) and placed in the dark. Images were taken 3 days after treatment. Horizontal bars indicate group median. **B,** Seedling hypocotyl length 3 days after treatment (*n* = 21–26 seedlings). Note that we failed to transform the LST8-1 loss-of-function mutant due to its severe phenotype. However, C-terminal fusion proteins of Arabidopsis LST8-1 have been published before^[1]^ and our results from *N. benthamiana* leaves confirmed that LST8-1::PafA successfully integrated into TORC. **C,** Wild type, RAPTOR1 loss-of function (*rb5*) and *rb5* complemented with the RAPTOR1 PUP-IT construct seedlings after 9 d of growth. **D,** Seedling root length after 9 d of growth (*n* = 11–22 seedlings). Bar height indicates group median. Different letters indicate statistically significant differences between groups based on an oneway ANOVA followed by a Tukey-HSD test (𝛼 = 0.05).


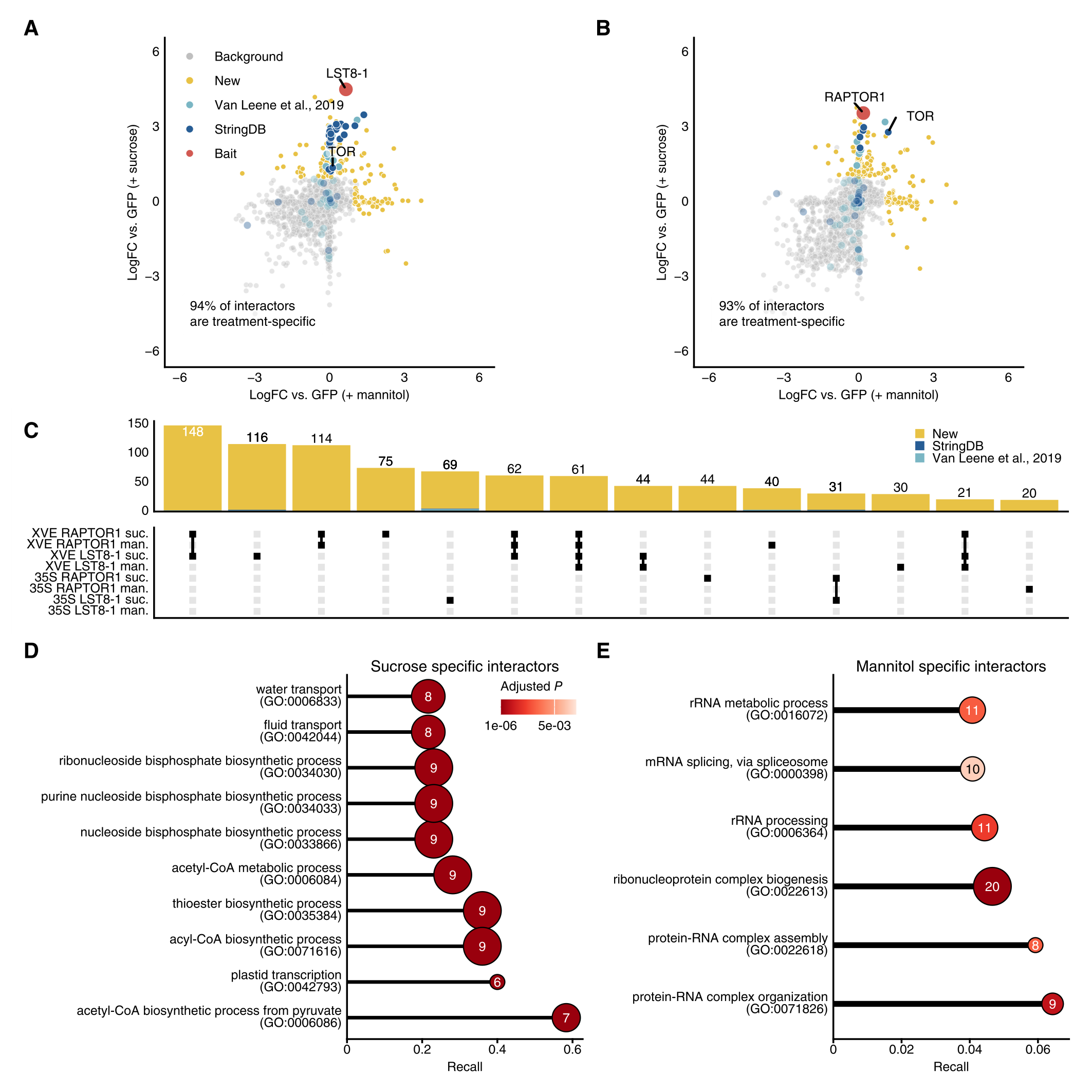


**Figure S3.** Inducible FLAG::PUP expression improves detection of TORC interactors across baits and treatments. **A–B,** Treatment-dependency of identified interactors using LST8-1 (**A**) or RAPTOR1 (**B**) baits and constitutive FLAG::PUP expression. Treatment- specific interactors are those only identified in one of the two treatments. Fold changes are calculated from *n* = 3 replicates using MsqRob2, *P*-values are corrected for multiple comparisons using the Benjamini-Hochberg FDR method. **C,** Upset plot showing overlaps in enriched proteins using the different used LST8-1 and RAPTOR1 constructs. The bars indicate the size of the exclusive overlap of the groups marked by black squares below. **D–E,** GO term enrichment for interactors specific to sucrose-treated and control seedlings, respectively.


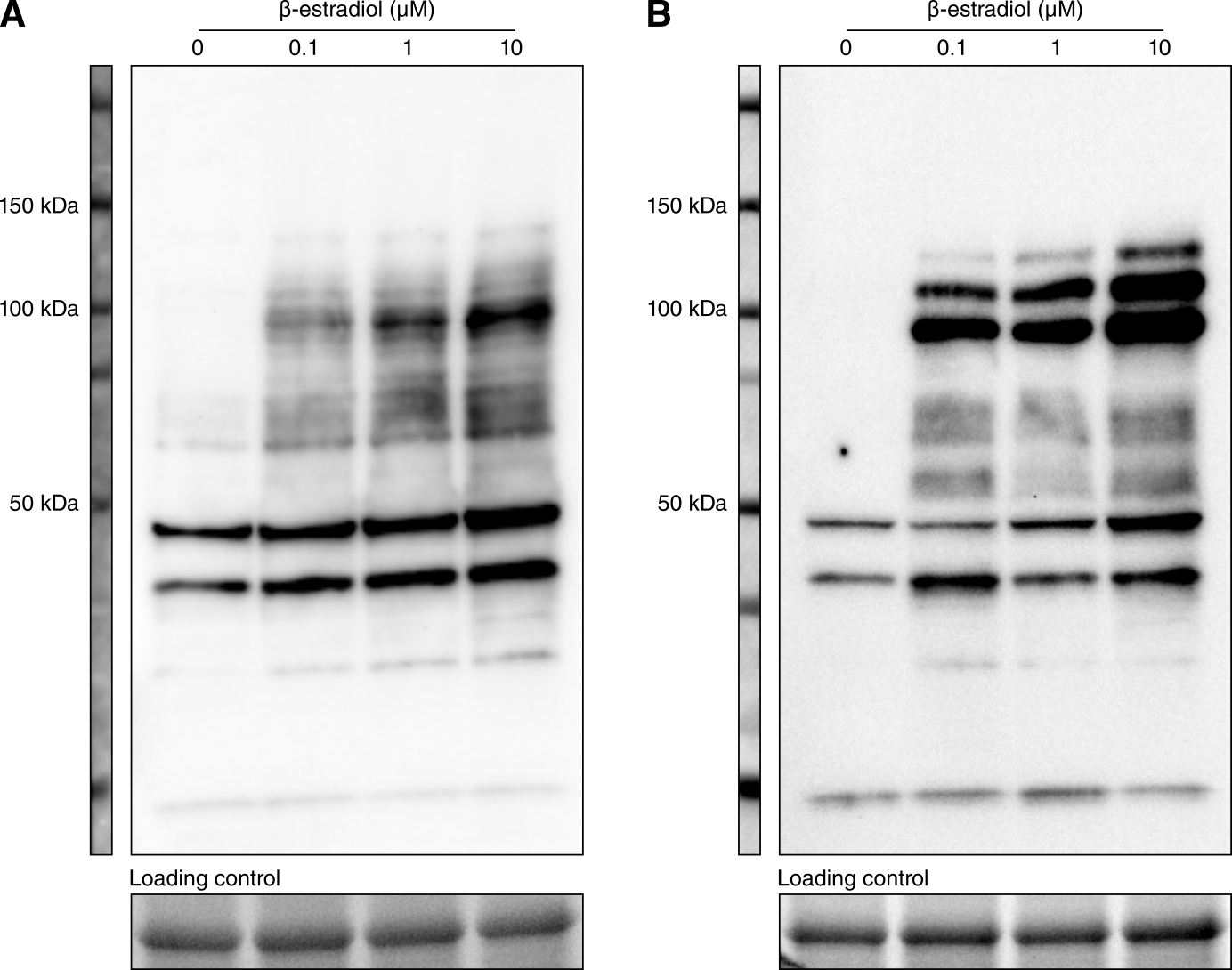


**Figure S4.** 𝛽-estradiol inducible XVE promoters show minimal bleed-through. Anti-FLAG Western blots of total protein extracted from seedlings carrying *N. benthamiana* leaves transiently expressing inducible constructs of LST8-1::PafA (**A**) and GFP::PafA (**B**), induced with varying concentrations of 𝛽-estradiol. The two strong bands below 50 kDa are unspecific signal. The loading control is Biorad ‘Stain-Free’ total protein fluorescence.


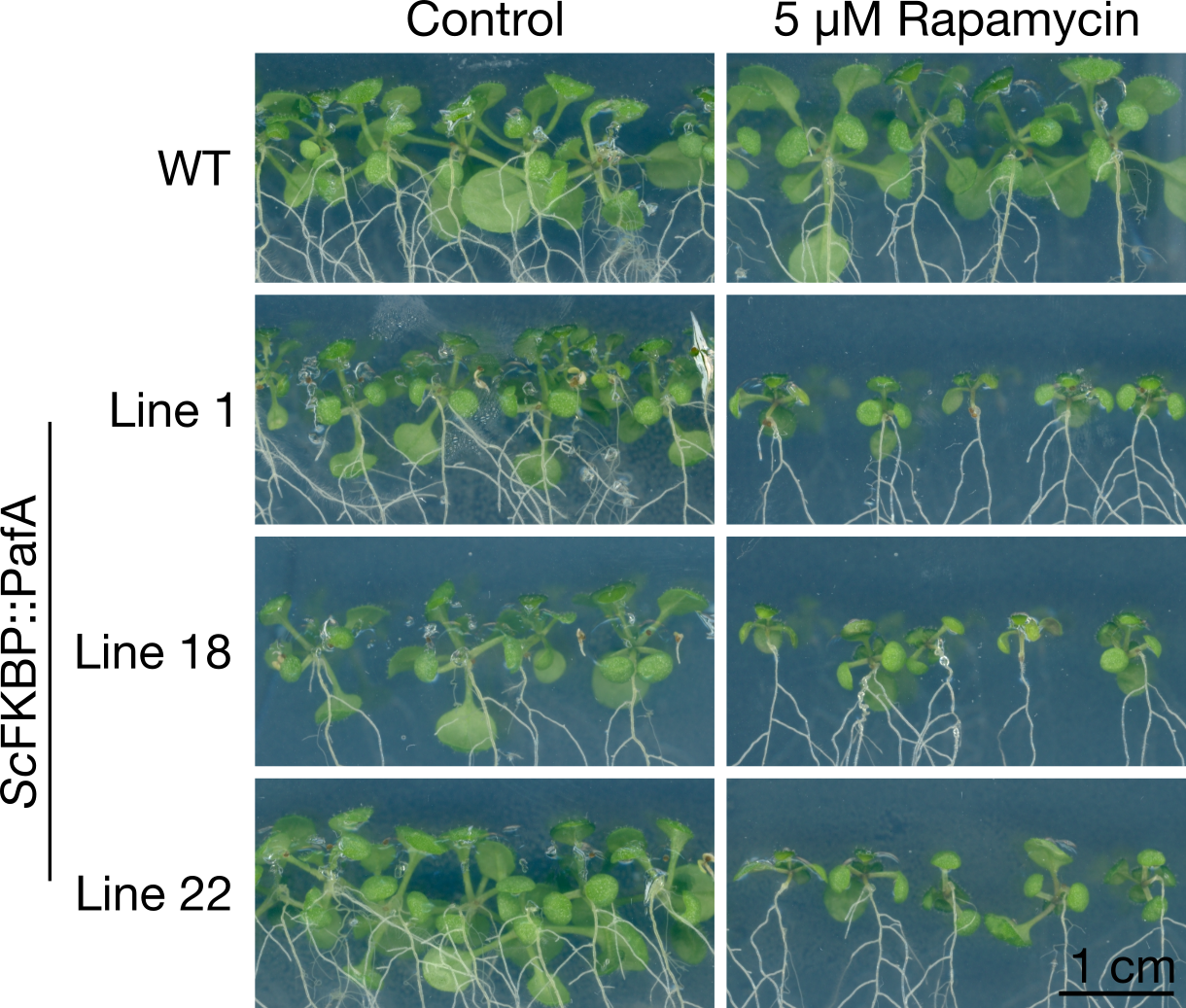


**Figure S5.** Rapamycin response in plants producing ScFKBP. Representative seedlings after 8 d of growth.


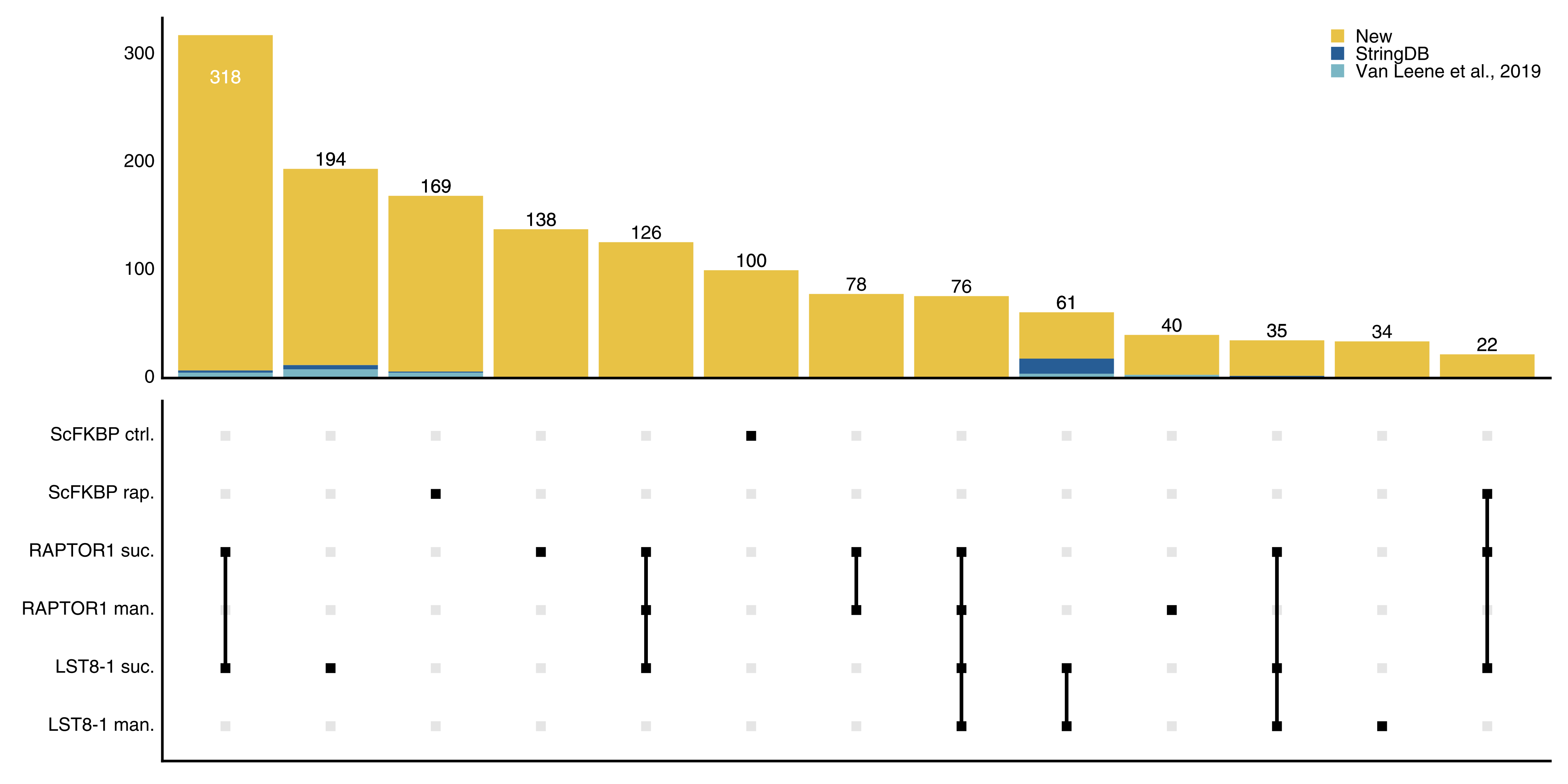


**Figure S6.** Overlap in TORC interactors identified with ScFKBP, LST8-1 and RAPTOR1 baits. The bars indicate the size of the exclusive overlap of the groups marked by black squares below.


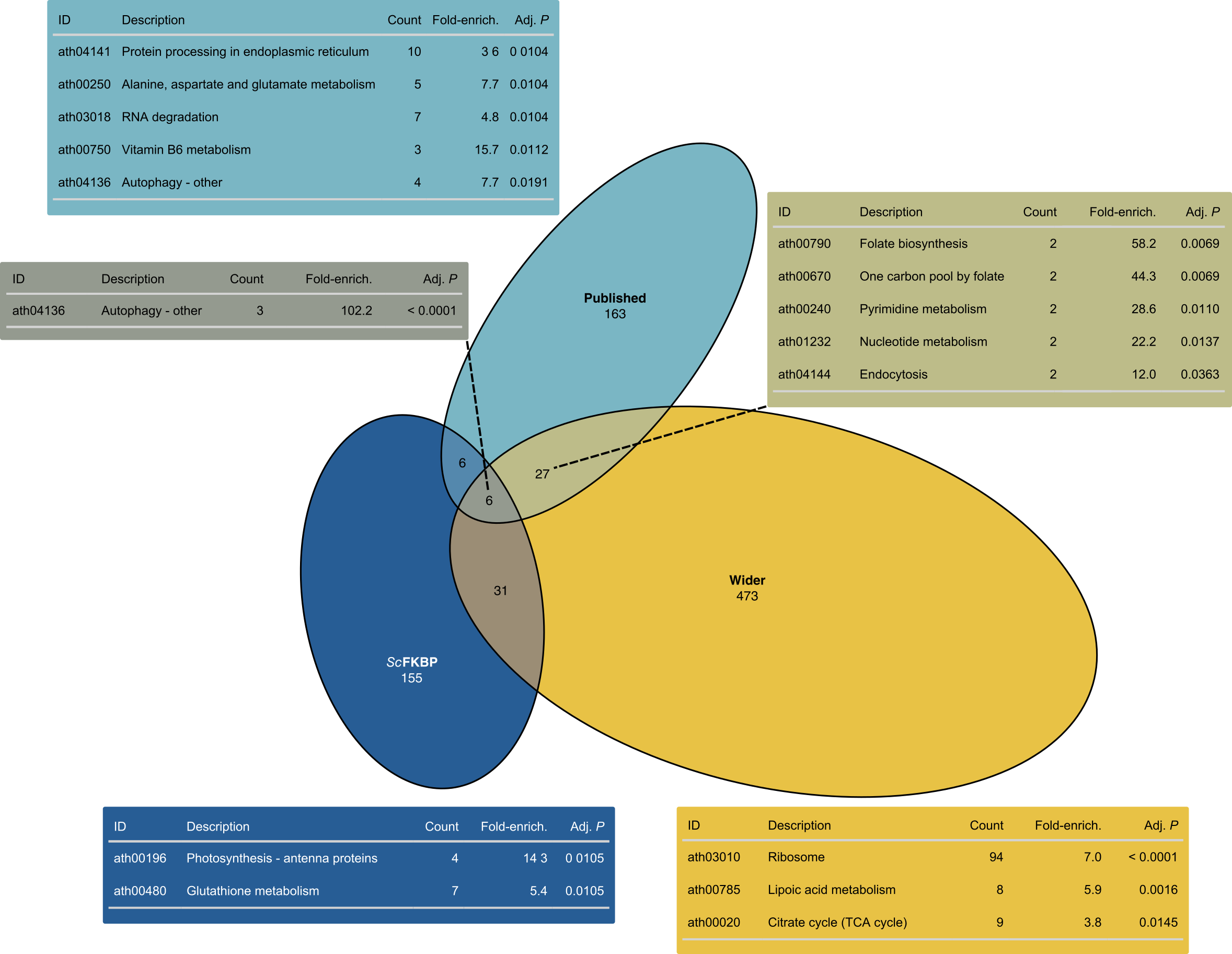


**Figure S7.** KEGG pathway enrichment of Published^[2,3]^ and TORC interactors identified in this study, as well as ScFKBP-specific interactors. Overlaps without an associated table did not yield any significantly over-represented terms.


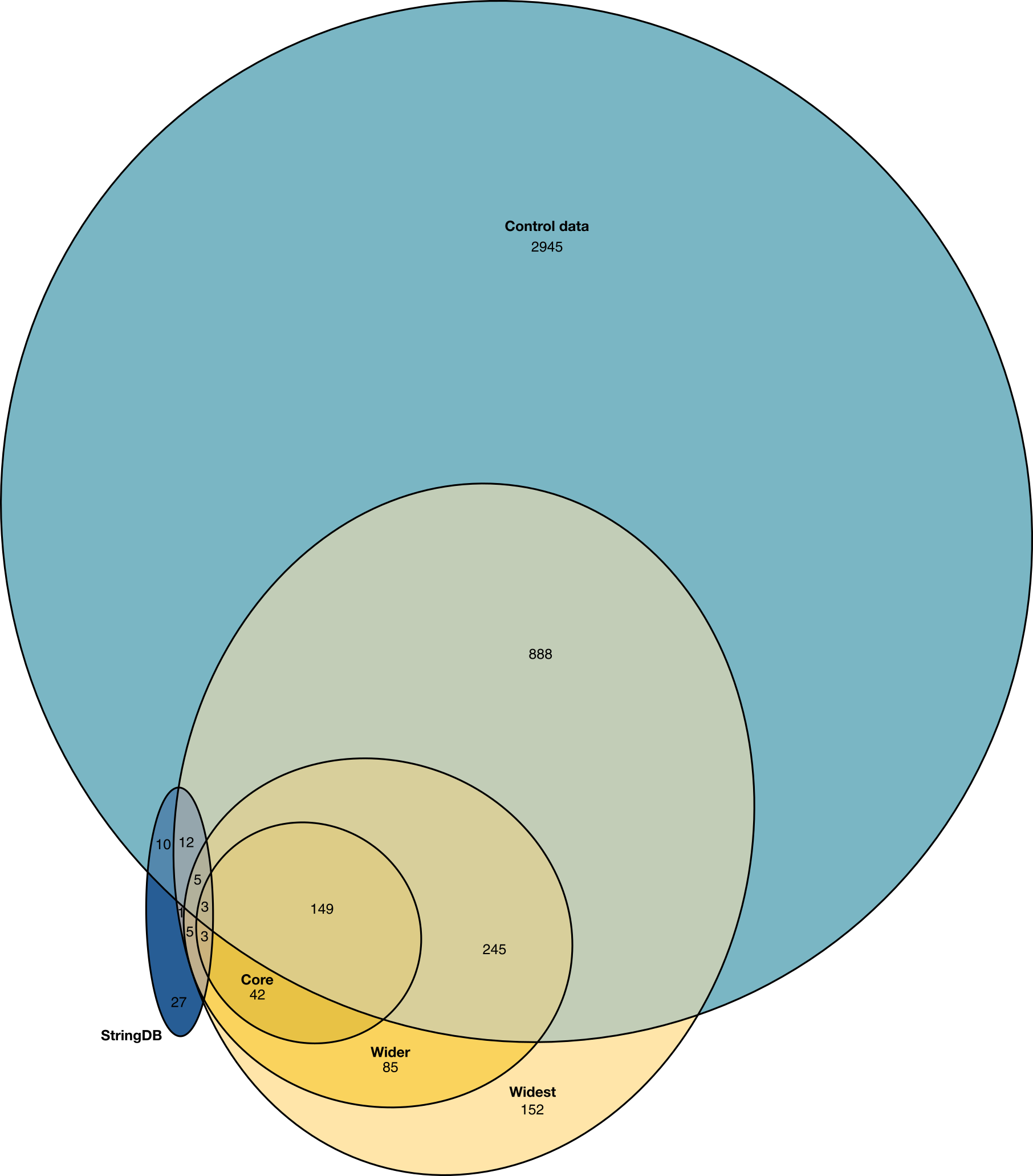


**Figure S8.** Overlap of the proposed TORC interactomes and previously published TORC interactors with proteins enriched in unrelated PUP-IT experiments. The control data includes proteins that were found enriched with baits not related to TOR or sugar signaling in separate experiments following the same experimental procedures. These proteins are therefore likely relatively promiscuous interactors, while the proteins found exclusively in our TOR experiments are more likely to be strictly involved in TOR signaling.


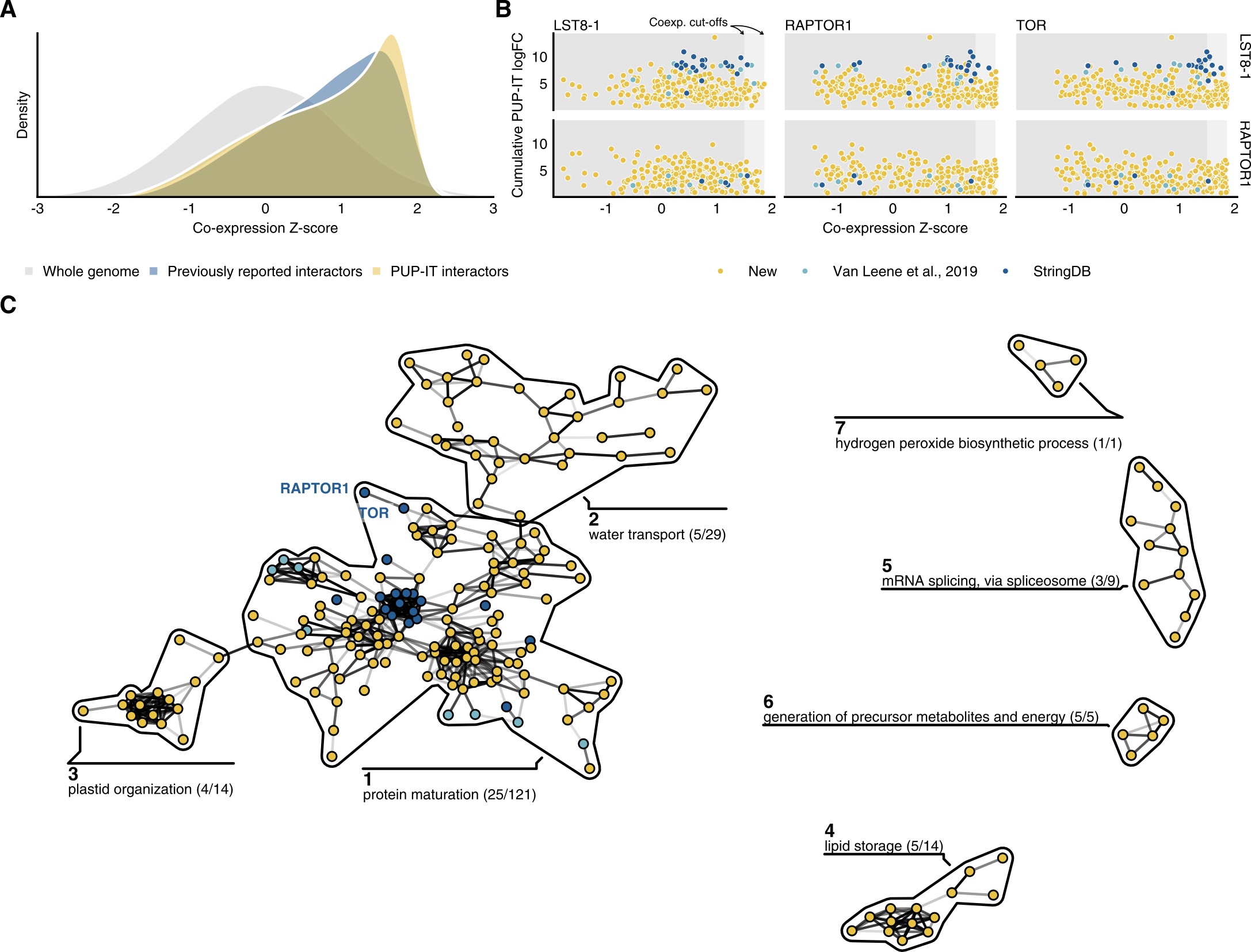


**Figure S9.** Co-expression analyses recover a subset of interactors identified with PUP- IT. **A,** Normalized co-expression scores of all pairwise combinations of genes within the Arabidopsis genome, previously reported TORC interactors in TORC interactors newly identified here using PUP-IT. **B,** Relationship between cumulative PUP-IT logFC values (i.e. the sums of logFC values from all independent experiments using the respective bait) and normalized co-expression score. Two appropriate thresholds for co-expression analyses are indicated by gray boxes, with 1.5 representing a generalist cut-off and 1.86 the 99th percentile of co-expression values observed among TORC interactors. Columns represent co-expression with the three TORC components, rows the cumulative logFC values with the two used TORC PUP-IT baits. **C,** Co-expression network of TORC interactors identified with PUP-IT. Edges are filtered to a normalized co-expression value greater than 1.86 (99th percentile), unconnected nodes or clusters composed of less than three nodes were trimmed. The most common GO biological process is indicated by cluster. TOR and RAPTOR1 are annotated, LST8-1 did not clear the co-expression threshold.


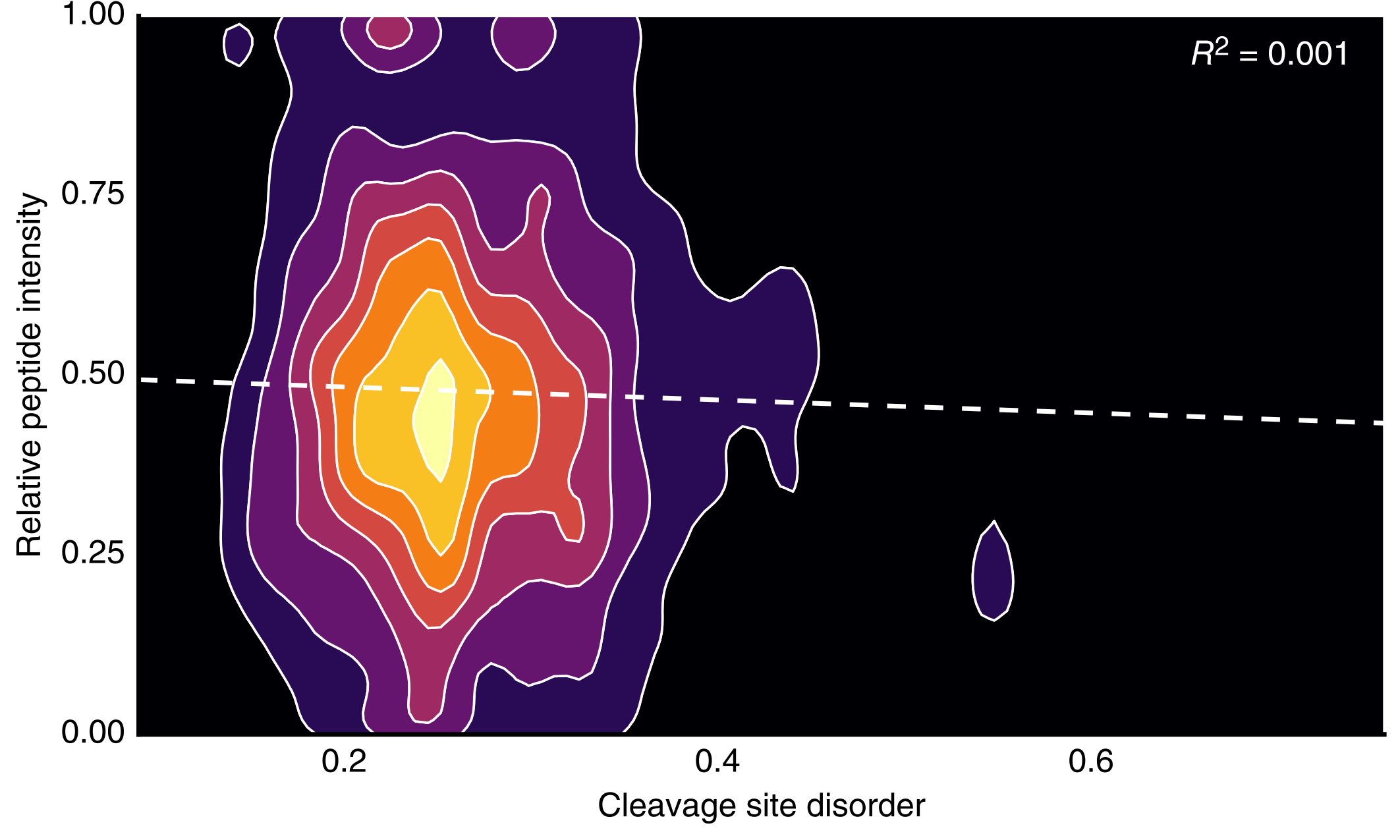


**Figure S10.** Local disorder does not affect peptide quantification by MS/MS. Of the proteins analyzed for lysine accessibility, those represented by more than 10 different peptides in the data from the inducible LST8-1 and RAPTOR1 constructs were selected. Log-transformed peptide intensities were range normalized for each protein (i.e. setting the most and least abundant peptide per protein to 1 and 0, respectively) and plotted against the average disorder of the peptide’s cleavage sites. To avoid overplotting of thousands of points, the values are represented as a density contour from black (lowest density) to yellow (highest density). The R^2^ of the linear regression is indicated.


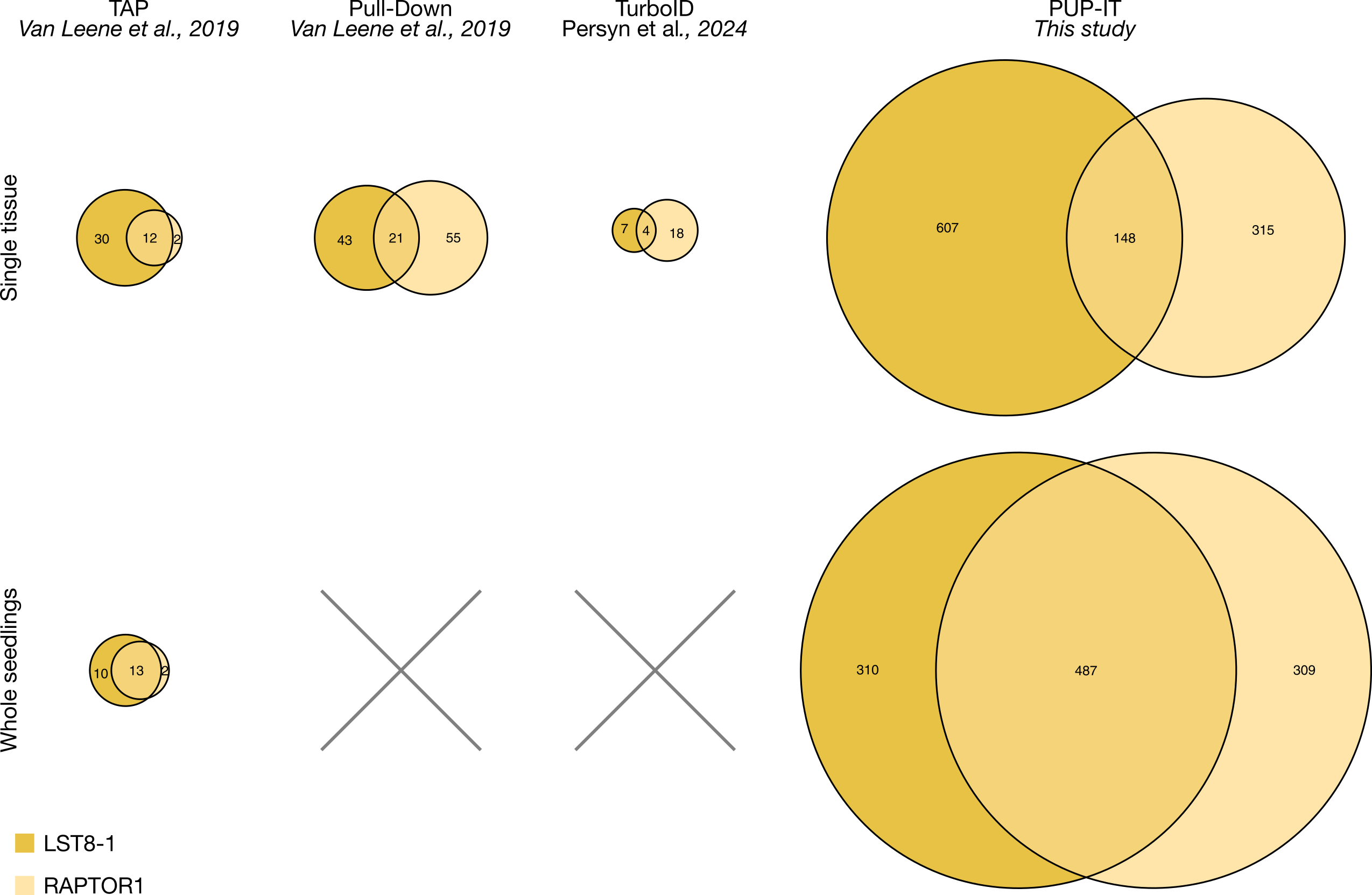


**Figure S11.** Consistency of results when using different TORC subunits as baits in pull-down, tandem-affinity purification or PUP-IT experiments.

# **Supplementary tables**

**Table S1. Primers used in the generation of PUP-IT constructs.**

Primer Sequence (5’->3’)

To generate entry clones that fuse bait proteins with PafA

| RAPTOR_F | agtttttctgattaacactagGTCGACATGGCATTAGGAGACTTAATGGTGTCT |
| --- | --- |
| RAPTOR_R | accagatcctcctccgccACTAGTTCTTGCTTGCGAGTTGTCGTGGGTGTATAT |
| LST8_F | agtttttctgattaacactagGTCGACATGAGTCAGCCTTCTGTGATTCTT |
| LST8_R | accagatcctcctccgccACTAGTATCGTGAAGTGCACAGCACACAGTGGCT |
| ScFKBP_F | agtttttctgattaacactagGTCGACATGTCTGAAGTAATTGAAGGTAAC |
| ScFKBP_R | accagatcctcctccgccACTAGTGTTGACCTTCAACAATTCGACGT |

To create destination vectors expressing FLAG::PUP(E) under β-estradiol inducible promoter

| XVE_F1 | ATGGAGCTCTCTAGAACTAGTagtctagctcaacagagcttttaacccaaattggt |
| --- | --- |
| XVE_R1 | cttataatccattgctagagtcgactagcttcagcgtgtcct |
| XVE_F2 | gactctagcaatggattataaggatgatgatgataaga |
| XVE_R2 | GTCGACGGTATCGATAAGCTTaccactttgtacaagaaagctgaacgagaa |

**Table S2. AlphaFold2-Multimer configuration.**

| Parameter | Version/Value |
| --- | --- |
| AlphaFold-Multimer | 2.3.1 |
| UniRef | 2023-12-18 |
| MGnify | 2023-02 |
| BFD | 2019-03 |
| UniProt | 2023-12-18 |
| PDB | 2023-12-18 |
| PDB70 | 22-03-13 |
| UniRef | –noali –F1 0.0005 –F2 5e-05 –F3 5e-07 –incE 0.0001 -E 0.0001 –cpu 2 -N 1 |
| MGnify | –noali –F1 0.0005 –F2 5e-05 –F3 5e-07 –incE 0.0001 -E 0.0001 –cpu 2 -N 1 |
| BFD | -n 3 -e 0.001 -maxseq 1000000 -realign_max 100000 -maxfilt 100000 -min_prefilter_hits 1000 |
| UniProt | –noali –F1 0.0005 –F2 5e-05 –F3 5e-07 –incE 0.0001 -E 0.0001 –cpu 2 -N 1 |
| PDB hmmbuild | –hand –amino |
| PDB hmmsearch | –noali –cpu 2 –F1 0.1 –F2 0.1 –F3 0.1 –incE 100 –E 100 –domE 100 –incdomE 100 |
| AFModelSettings | -g true -r false -e false -m multimer -c full_dbs -p true -l 1 -b false -d -t ’1950-01-01’ |

**Table S3. Numbers of interactors found in individual experiments.**

| Bait | Expression | Treatment | Time | Total | StringDB | Van Leene et al., 2019 |
| --- | --- | --- | --- | --- | --- | --- |
| LST8-1 | Constitutive | Mannitol | 24 h | 65 | 2 | 3 |
| LST8-1 | Constitutive | Sucrose | 24 h | 198 | 24 | 38 |
| RAPTOR1 | Constitutive | Mannitol | 24 h | 79 | 1 | 2 |
| RAPTOR1 | Constitutive | Sucrose | 24 h | 154 | 6 | 11 |
| LST8-1 | Inducible | Mannitol | 24 h | 114 | 16 | 20 |
| LST8-1 | Inducible | Mannitol | 4 h | 149 | 18 | 24 |
| LST8-1 | Inducible | Sucrose | 24 h | 160 | 21 | 26 |
| LST8-1 | Inducible | Sucrose | 4 h | 499 | 21 | 32 |
| RAPTOR1 | Inducible | Mannitol | 24 h | 199 | 3 | 4 |
| RAPTOR1 | Inducible | Mannitol | 4 h | 256 | 2 | 3 |
| RAPTOR1 | Inducible | Sucrose | 24 h | 187 | 2 | 3 |
| RAPTOR1 | Inducible | Sucrose | 4 h | 480 | 2 | 6 |
| ScFKBP | Constitutive | Rapamycin | 24 h | 198 | 5 | 10 |
| ScFKBP | Constitutive | Rapamycin | 4 h | 74 | 0 | 3 |

**Table S4. Proteins carrying the identified Phosphomotifs.**

| Gene | Protein | Position | Description | Window |
| --- | --- | --- | --- | --- |
| ’SP’ motif |  |  |  |  |
| AT3G45780 | O48963 | 350 | phototropin 1 | RPRALS[79.9663]ESTNL |
| AT2G29210 | Q8L7W3 | 444 | splicing factor PWI domain-containing protein | GARLPS[79.9663]PSIEQ |
| AT3G20550 | Q8W4D8 | 133 | SMAD/FHA domain-containing protein | NARGGS[79.9663]EEPNV |
| AT1G42550 | Q9C8E6 | 328 | plastid movement impaired1 | SFSVPS[79.9663]PKMTS |
| AT1G59610 | Q9LQ55 | 719 | dynamin-like 3 | MRQSLS[79.9663]EGSLD |
| AT3G17840 | Q9LVI6 | 331 | receptor-like kinase 902 | YVNEYS[79.9663]PSAVK |
| AT3G02880 | Q9M8T0 | 621 | Leucine-rich repeat protein kinase family protein | SHSSGS[79.9663]PNPVS |
| AT3G08850 | Q93YQ1 | 916 | Regulatory-associated protein of TOR 1 | RTPPVS[79.9663]PPRTN |
| AT4G27450 | Q93V62 | 219 | aluminum induced protein with YGL and LRDR motifs | MPRVDS[79.9663]EGVLC |
| AT5G57110 | Q9LF79 | 8 | autoinhibited Ca2+ -ATPase | SLLKSS[79.9663]PGRRR |
| AT1G59610 | Q9LQ55 | 533 | dynamin-like 3 | LNRATS[79.9663]PQPDG |
| AT1G59610 | Q9LQ55 | 844 | dynamin-like 3 | SGTESS[79.9663]PRTNG |
| AT1G10290 | Q9SE83 | 533 | dynamin-like protein 6 | LSRATS[79.9663]PQPDG |
| AT1G10290 | Q9SE83 | 837 | dynamin-like protein 6 | SGTESS[79.9663]PRASG |
| AT3G48740 | Q9SMM5 | 248 | Nodulin MtN3 family protein | LGTVSS[79.9663]PEPIS |
| AT3G15450 | Q9LE80 | 218 | aluminum induced protein with YGL and LRDR motifs | MPRIDS[79.9663]EGVLC |
| AT5G22650 | Q56WH4 | 266 | histone deacetylase 2B | VNANQS[79.9663]PKSGG |
| ’RxxS’ motif | |  |  |  |
| AT3G45780 | O48963 | 350 | phototropin 1 | RPRALS[79.9663]ESTNL |
| AT4G24275 | Q84R17 | 44 | — | LSRNRS[79.9663]VSASA |
| AT2G29210 | Q8L7W3 | 444 | splicing factor PWI domain-containing protein | GARLPS[79.9663]PSIEQ |
| AT3G20550 | Q8W4D8 | 133 | SMAD/FHA domain-containing protein | NARGGS[79.9663]EEPNV |
| AT1G19870 | Q9FXI5 | 706 | IQ-domain 32 | SGRRTS[79.9663]FGYDQ |
| AT5G10360 | P51430 | 241 | Ribosomal protein S6e | RSRLSS[79.9663]APAKP |
| AT1G59610 | Q9LQ55 | 823 | dynamin-like 3 | LTRQLS[79.9663]IHDNR |
| AT4G32180 | Q8L5Y9 | 46 | pantothenate kinase 2 | IHRSGS[79.9663]RPQLD |
| AT4G27450 | Q93V62 | 219 | aluminum induced protein with YGL and LRDR motifs | MPRVDS[79.9663]EGVLC |
| AT5G43830 | Q9FG81 | 216 | aluminum induced protein with YGL and LRDR motifs | VPRVDS[79.9663]SGDVC |
| AT5G43830 | Q9FG81 | 243 | aluminum induced protein with YGL and LRDR motifs | MPRVDS[79.9663]SQNWA |
| — | Q9FKA5 | 337 | — | QRRNRS[79.9663]GSGDD |
| AT1G59610 | Q9LQ55 | 533 | dynamin-like 3 | LNRATS[79.9663]PQPDG |
| AT1G10290 | Q9SE83 | 533 | dynamin-like protein 6 | LSRATS[79.9663]PQPDG |
| AT3G15450 | Q9LE80 | 218 | aluminum induced protein with YGL and LRDR motifs | MPRIDS[79.9663]EGVLC |
| AT3G58730 | Q9XGM1 | 241 | vacuolar ATP synthase subunit D (VATD) | MQRGIS[79.9663]INAAR |
| — | Q9FKA5 | 339 | — | RNRSGS[79.9663]GDDEE |

# **Supplementary bibliography**

1. [M. Moreau, M. Azzopardi, G. Clément, T. Dobrenel, C. Marchive, C. Renne, M.-L. Martin-Magniette, L. Taconnat, J.-P. Renou, C. Robaglia, C. Meyer,](https://doi.org/10.1105/tpc.111.091306) [Plant Cell](https://doi.org/10.1105/tpc.111.091306) [2012,](https://doi.org/10.1105/tpc.111.091306) [24, 463.](https://doi.org/10.1105/tpc.111.091306)
2. [J. Van Leene, C. Han, A. Gadeyne, D. Eeckhout, C. Matthijs, B. Cannoot, N. De Winne, G. Persiau, E. Van De Slijke, B. Van de Cotte, E. Stes, M. Van Bel, V. Storme, F. Impens, K. Gevaert, K. Vandepoele, I. De Smet, G. De Jaeger,](https://doi.org/10.1038/s41477-019-0378-z) [Nat. Plants 2019,](https://doi.org/10.1038/s41477-019-0378-z) [5, 316](https://doi.org/10.1038/s41477-019-0378-z).
3. [F. Persyn, W. Smagghe, D. Eeckhout, T. Mertens, T. Smorscek, N. De Winne, G. Persiau, E. Van De Slijke, N. Crepin, A.](https://doi.org/10.1016/j.mcpro.2024.100842) [Gadeyne, J. Van Leene, G. De Jaeger,](https://doi.org/10.1016/j.mcpro.2024.100842) [Mol. Cell. Proteomics](https://doi.org/10.1016/j.mcpro.2024.100842) [2024, 100842.](https://doi.org/10.1016/j.mcpro.2024.100842)
